# The Small Interactor of PKD2 protein promotes the assembly and ciliary entry of the *Chlamydomonas* PKD2–mastigoneme complexes

**DOI:** 10.1242/jcs.261497

**Published:** 2024-01-12

**Authors:** Poulomi Das, Betlehem Mekonnen, Rama Alkhofash, Abha V. Ingle, E. Blair Workman, Alec Feather, Gui Zhang, Nathan Chasen, Peiwei Liu, Karl F. Lechtreck

**Affiliations:** ^1^ Department of Cellular Biology, University of Georgia, Athens, GA 30602, USA; ^2^ Department of Computer Science, University of Georgia, Athens, GA 30602, USA

**Keywords:** TRP channel, Cilia, Intraflagellar transport

## Abstract

In *Chlamydomonas*, the channel polycystin 2 (PKD2) is primarily present in the distal region of cilia, where it is attached to the axoneme and mastigonemes, extracellular polymers of MST1. In a smaller proximal ciliary region that lacks mastigonemes, PKD2 is more mobile. We show that the PKD2 regions are established early during ciliogenesis and increase proportionally in length as cilia elongate. In chimeric zygotes, tagged PKD2 rapidly entered the proximal region of PKD2-deficient cilia, whereas the assembly of the distal region was hindered, suggesting that axonemal binding of PKD2 requires *de novo* assembly of cilia. We identified the protein Small Interactor of PKD2 (SIP), a PKD2-related, single-pass transmembrane protein, as part of the PKD2–mastigoneme complex. In *sip* mutants, stability and proteolytic processing of PKD2 in the cell body were reduced and PKD2–mastigoneme complexes were absent from the cilia. Like the *pkd2* and *mst1* mutants, *sip* mutant cells swam with reduced velocity. Cilia of the *pkd2* mutant beat with an increased frequency but were less efficient in moving the cells, suggesting a structural role for the PKD2–SIP–mastigoneme complex in increasing the effective surface of *Chlamydomonas* cilia.

## Teaser

**Summary:** Small Interactor of PKD2 (SIP) is a novel, single-transmembrane-helix PKD2-related protein that is a crucial part of the ciliary PKD2–mastigoneme complex in *Chlamydomonas*.

## INTRODUCTION

Cilia and eukaryotic flagella are microtubule-based cell projections with motile and sensory functions. The latter involves channels and receptors in the ciliary membrane, which covers the ciliary axoneme and is continuous with the plasma membrane. Rather than being homogenous in composition, the ciliary membrane often contains sub-compartments in which specific membrane proteins are concentrated. In the auditory cilia of *Drosophila* chordotonal neurons, for example, the TRP channel NompC is localized in parts of the distal zone whereas voltage-gated TRPV channels are present in the proximal zone (Xiang et al., 2022). Also in *Drosophila*, the polycystin 2 (PKD2) ortholog AMO is located near the tip of sperm cilia (Köttgen et al., 2011; Watnick et al., 2003). Similarly, the olfactory cyclic nucleotide-gated channel subunit 1 (OcNC1; also known as Cnga2) is concentrated in the distal segments of rat olfactory cilia (Matsuzaki et al., 1999). The salt-sensing receptor guanylate cyclase GCY-22 also resides in the distal region of *Caenorhabditis elegans* primary cilia of ASER neurons (van der Burght et al., 2020). In the latter, the localization of GCY-22 requires motor-driven intraflagellar transport (IFT), a protein shuttle dedicated to the assembly and maintenance of cilia, to continuously capture the receptor along the length of cilia and return it to the tip by anterograde IFT. Thus, proteins can be confined to certain ciliary regions dynamically by active transport. However, other membrane protein patterns are more static, which is likely to involve anchoring of membrane proteins to underlying axonemal structures. Indeed, NompC attaches via its ankyrin repeat domain to the underlying microtubules, forming a spring-like connection, which contributes to mechanical gating of the channel (Zhang et al., 2015). In addition to patterns along the proximo-distal axis, some membrane proteins assume additional levels of order around the circumference of cilia. An example is the multiprotein channel complex CatSper, which forms four intricately patterned rows, the race stripes, along the principal piece of mammalian sperm flagella (Chung et al., 2014; Zhao et al., 2022). The observations raise numerous questions, including how such membrane proteins are targeted to their specific positions within cilia, how the length of the specialized membrane subdomains is determined and how these regions scale with respect to the overall length of cilia. Another open question is what role such membrane protein patterns play in the variety of motile and sensory functions exhibited by cilia across species and cell types.

To start addressing these questions, we analyzed the distribution of the TRP channel PKD2 in *Chlamydomonas*, a tractable system for the genetic, biochemical, microscopic and functional analysis of cilia (Dutcher, 1995; Lechtreck, 2016; Pazour et al., 2005; Silflow and Lefebvre, 2001). In mammals, PKD1 and PKD2 form a 1:3 complex and the proteins are present on cilia in the kidney and the embryonic node (Pennekamp et al., 2002; Su et al., 2018). Mutations in either protein cause autosomal dominant polycystic kidney disease (Wu and Somlo, 2000). PKD2 also participates in the determination of the left–right body axis, likely by sensing the flow generated by the motile monocilia in the center of the murine embryonic node. In the non-motile nodal cilia, PKD2 is preferentially located on the dorsal side, facing the flow (Katoh et al., 2023). This localization supports the idea that a specific position within cilia or orientation with respect to the flow could contribute to PKD2 function. In *C. elegans*, the polycystin homologs LOV-1 and PKD-2 (also known as PC1 and PC2, respectively) are expressed specifically in male-specific sensory neurons; both proteins localize to primary cilia, and are required for male mating behavior (Barr and Sternberg, 1999; Walsh et al., 2022). PKD2 is also present in non-metazoans, which typically lack PKD1 homologs, raising questions about the composition and function of PKD2 channel complexes in those species (Liu et al., 2023). In fission yeast, a PKD2-like protein, which possesses nine transmembrane helices, is located in the plasma membrane, contributing to cellular Ca^2+^ homeostasis. It has been proposed that this protein is responsible for sensing membrane tension during cytokinesis (Poddar et al., 2022). In *Chlamydomonas*, PKD2 is cleaved within the large extracellular loop between transmembrane helix 1 and 2 via a currently unknown mechanism (Huang et al., 2007). Cleavage occurs in the cell body, and the two PKD2 fragments enter cilia, remaining associated to each other (Huang et al., 2007; Liu et al., 2020). Inside cilia, *Chlamydomonas* PKD2 attaches, directly or indirectly, to the axonemal doublet microtubules (DMTs) 4 and 8 and is required for anchoring the mastigonemes, thread-like extracellular polymers of the glycoprotein MST1, to the ciliary surface (Liu et al., 2023, 2020). The ultrastructure of MST1 was recently determined (PDB: 8TX1) and is dominated by immunoglobulin-like and Sushi domains, structural elements observed in many extracellular proteins (Wang et al., 2023). On the cilia, the mastigoneme rows are oriented perpendicular to the plane of the ciliary beat, generating a fan-like structure (Nakamura et al., 1996; Witman et al., 1972). This arrangement should increase the effective surface of the cilium, affecting the swimming velocity of the cell (Liu et al., 2020; Nakamura et al., 1996). However, the phenotype of PKD2- and MST1-deficient cells is subtle and the swimming velocity of mastigoneme-deficient cells has been analyzed repeatedly with conflicting outcomes (Amador et al., 2020; Liu et al., 2020; Nakamura et al., 1996; Wang et al., 2023). In addition to the stationary PKD2–mastigoneme complexes in the distal region of *Chlamydomonas* cilia, PKD2 without attached mastigonemes is present in a separate proximal region of cilia; here, PKD2 is more mobile, moving by slow diffusion (Liu et al., 2020). This raises the question of how cells sort and assemble PKD2 into two distinct ciliary domains.

Here, we analyzed how the distribution of PKD2 in *Chlamydomonas* is established during both ciliary assembly and the repair of PKD2-deficient full-length cilia, and how this distribution is linked to ciliary length. Furthermore, we identified the protein Small Interactor of PKD2 (SIP), a novel single-pass transmembrane protein, related to the N-terminal portion of PKD2. Cilia of the *sip* mutant lack PKD2–mastigoneme complexes and swim with reduced velocity. In the *sip* mutant, stability and proteolytic processing of PKD2 in the cell body were strongly reduced, suggesting that SIP contributes to PKD2 complex formation, processing and ciliary entry.

## RESULTS

### The two PKD2 regions are established early during cilia regeneration

**Fig. 1.**
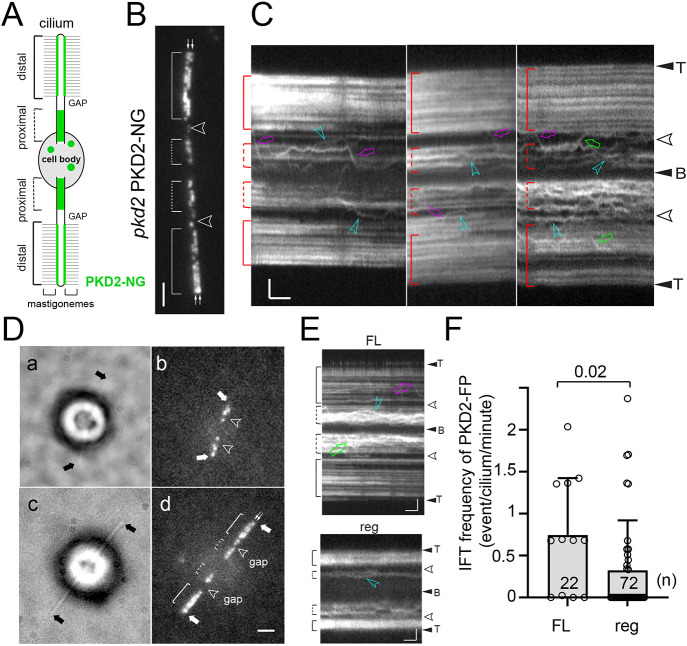
**PKD2 regions develop early during ciliogenesis.** (A) Schematic representation of an adhered *Chlamydomonas* cell. The mastigonemes and PKD2–NG (green) are indicated. Green circles indicate the pool of PKD2–NG in the apical region of the cell body as shown in Fig. S1A. (B) TIRF image of *pkd2 PKD2-NG* cilia. The distal and proximal PKD2–NG regions are marked with brackets. Open arrowheads indicate the gap regions. Small arrows indicate the rows of PKD2-NG. Scale bar: 2 µm. (C) Kymograms of full-length *pkd2* PKD2-NG cilia. The ciliary tips (T), bases (B), the distal and proximal regions, and gaps are marked. Magenta arrows, retrograde IFT; green arrows, anterograde IFT; blue arrowheads, apparent diffusion of PKD2-NG. Scale bars: 2 s and 2 µm. (D) Bright field (a,c) and TIRF (b,d) images of *pkd2 PKD2-mNG* cells with regenerating cilia. Arrowheads indicate the gap and single arrows indicate the ciliary tips. The distal and proximal PKD2–NG regions and the intercalated gap are marked with brackets and arrowheads. Small double arrows in d indicate the two rows of PKD2-NG. Scale bars: 2 µm. (E) Kymograms of full-length (FL) and regenerating (reg) *pkd2* PKD2–NG cilia. The ciliary tips (T), bases (B), the distal and proximal regions, and gaps are marked. Magenta arrow, retrograde IFT; green arrow, anterograde IFT; blue arrowheads, apparent diffusion of PKD2-NG. Scale bars: 2 s and 2 µm. (F) Combined anterograde and retrograde IFT frequency of PKD2-NG in full length (FL) and regenerating (reg) *pkd2 PKD2-NG* cilia. Error bars show the s.d.; the number of cilia analyzed (*n*) and the result of a two-tailed unpaired *t*-test are indicated. Images in B–E are representative of 20 or more repeats.

**Fig. 2.**
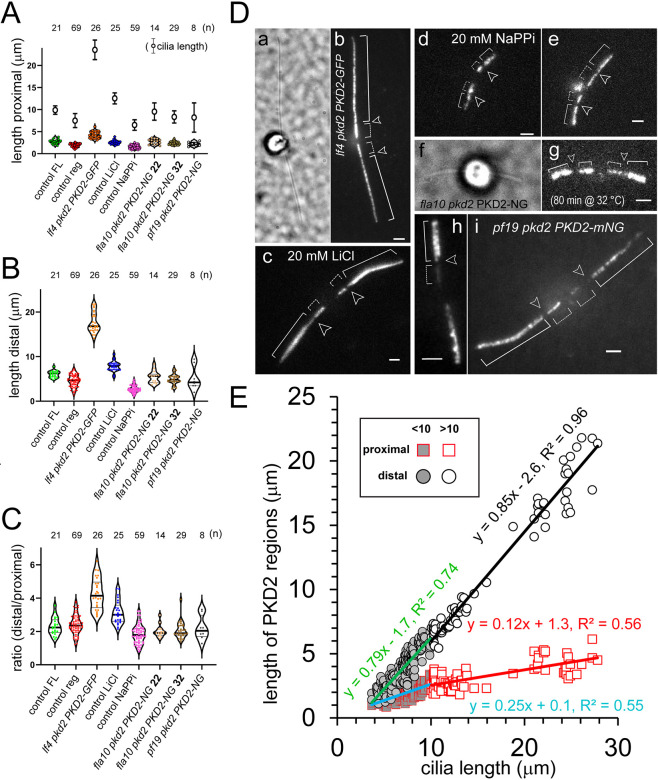
**PKD2 regions adjust in length in short cilia and the distal region is extended on long cilia.** (A–C) Violin plots showing the lengths of the proximal region (A), the length the distal region (B), and the ratios between the two (C) in full-length (FL, green) and regenerating (reg, red) *pkd2 PKD2-NG* cilia (control), cilia of the *lf4 pkd2 PKD2-GFP* strain (orange), *pkd2 PKD2-NG* cilia of cells treated with LiCl (blue) or NaPPi (magenta), *fla10 pkd2 PKD2-NG* cilia at the permissive (22°C, light brown) and restrictive (32°C, dark brown) temperatures, and cilia of a *pf19 pkd2 PKD2-NG* double mutant (gray). The number of cilia analyzed is indicated; the average length and the s.d. of the cilia are indicated in A. (D) Bright field (a,f) and TIRF images (b–e and g–i) of a *lf4 pkd2 PKD2-GFP* cell (a,b), a cell treated with 20 mM LiCl (c), cells treated with 20 mM NaPPi (d,e), a *fla10 pkd2 PKD2-NG* cell after 80 min incubation at 32°C (f,g) and two *pf19 pkd2 PKD2-NG* cells (h,i). The gap (arrowheads) and the PKD2–NG regions (brackets) are marked. Scale bars: 2 µm. (E) Summary plot of the length of the proximal (squares) and distal (circles) PKD2–NG region lengths versus ciliary length. Trend lines were calculated for cilia shorter than 10 µm (blue and red) and longer than 10 µm (green and black).

In full-length *Chlamydomonas* cilia, PKD2 is organized into two distinct regions: an ∼2.7 µm long region occupying the proximal part of the cilia, in which PKD2 fused at its C-terminus to mNeonGreen (PKD2–NG) moves by slow diffusion and which does not contain mastigonemes, and the distal region of ∼6 µm length, in which PKD2 forms two more-or-less irregular rows anchoring the mastigonemes to the ciliary surface (Fig. 1A,B; Fig. S1A; Table 1) (Liu et al., 2020). The PKD2–NG rows are not always clearly discernable in our micrographs because their distance of ∼200 nm is near the limit of resolution of standard light microscopy (DeCaen et al., 2013). The proximal and distal PKD2–NG regions are typically (in >90% of cilia) separated by a more or less conspicuous gap of ∼1 µm length lacking PKD2 but for the occasional particle passing through by IFT or diffusion (Fig. 1C; Fig. S1A). Furthermore, PKD2–NG was present in a punctate pool in the apical region of the cells (Fig. S1Ad,e). To study how the PKD2 regions develop during cilia formation, *pkd2* PKD2–NG cells were de-ciliated by a pH shock and analyzed at various time points during cilia regeneration using *in vivo* total internal reflection fluorescence microscopy (TIRFM). The distribution of PKD2–NG in a proximal and distal region with a gap separating the two was apparent in most of the short regenerating cilia that were analyzed; in a subset of cells, PKD2–NG was absent or sparse within the proximal region of short regenerating cilia (Fig. 1D). The ratio between the proximal and distal PKD2–NG region in full-length and regenerating cilia was 2.3 and 2.4, respectively (Fig. 2A–C, Table 1). Thus, the distribution of PKD2–NG into two compartments is established early during cilia regeneration and the length of both regions increases proportionally relative to ciliary length. The plot in Fig. S2A shows that the proximal border of the distal PKD2–NG region shifts away from the ciliary base as cilia grow and the proximal region expands, suggesting continued remodeling of anchored PKD2–NG as cilia elongate.

**Table 1.**
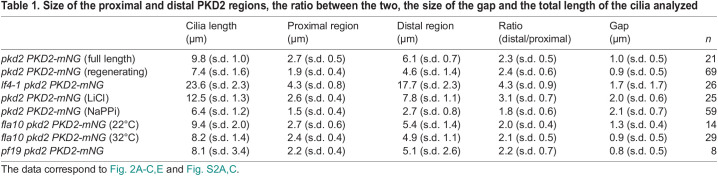
Size of the proximal and distal PKD2 regions, the ratio between the two, the size of the gap and the total length of the cilia analyzed

In *Chlamydomonas*, PKD2 is a confirmed cargo of IFT, but IFT of PKD2 is not observed in *C. elegans* (Huang et al., 2007; Liu et al., 2020; Qin et al., 2005). *In vivo* imaging showed low IFT frequencies of *Chlamydomonas* PKD2–NG in both full-length (i.e. cilia of cells not treated by a pH shock) and regenerating cilia, (0.81 events/min versus 0.35 events/min, respectively; Fig. 1E,F) (Huang et al., 2007; Liu et al., 2020). PKD2–NG was, however, more abundant in full-length cilia, which could result in more frequent transports via IFT. Given that IFT of PKD2–NG is not upregulated during ciliary regeneration (as it has been described for many axonemal proteins; Lechtreck, 2022), this suggests that the majority of PKD2–NG enters the ciliary compartment through an IFT-independent process.

### The two PKD2 regions maintain a similar ratio in abnormally short, but not abnormally long, cilia

Next, we tested whether the distribution of PKD2 is affected by perturbations of ciliary length using genetic or pharmacological means. First, we expressed PKD2–GFP in *lf4* cells, a mutant that assembles cilia exceeding the length of control cilia by up to three times owing to the lack of the CDK-related kinase LF4 (also known as MOK) (Berman et al., 2003) (Fig. 2Da,b). In *lf4 pkd2* PKD2–GFP cilia, we observed a pronounced elongation of the distal region (∼17.7 µm) whereas the proximal region extended only moderately (∼4.3 µm), leading to the length ratio of on average of ∼1:4.3 between the two regions (Fig. 2A–C, Table 1). Whole-mount electron microscopy (EM) of negative stained *lf4* cells showed that mastigonemes are present along the most parts of the long cilia, supporting the notion that the distal mastigoneme-carrying region of PKD2 is enlarged (Fig. S2B). Next, we treated *pkd2* PKD2–NG cells with 20 mM LiCl, which induces ciliary elongation (Nakamura et al., 1987; Wilson and Lefebvre, 2004). After incubation in medium supplemented with 20 mM LiCl for ∼60 min, cilia were elongated (12.5 μm compared to 9.8 μm for untreated cells) and the average ratio between the distal and proximal PKD2–NG region had readjusted to ∼3.1 (Fig. 2A–C,Dc, Table 1). These data indicate that the distal region particularly increases in cilia that are longer than normal. To determine how the distribution of PKD2 is affected when cilia are shortened, we treated *pkd2* PKD2–NG cells with sodium pyrophosphate (NaPPi) to induce cilia resorption (Lefebvre et al., 1978). After a 60–120-min incubation in 20 mM NaPPi, most cells (∼90%) had partially resorbed their cilia to an average length of ∼6.4 μm (Fig. 2A-C,Dd,e, Table 1). Although we observed variations between cells, both the distal and proximal PKD2–NG region decreased in length (∼2.7 and 1.5 µm, respectively), resulting in an average ratio of ∼1.8 (Fig. 2C). This suggests that NaPPi-induced ciliary resorption does not occur by simple shortening from the tip but involves reorganization of PKD2–NG. *Chlamydomonas* cilia also shorten in the absence of active IFT (Kozminski et al., 1995; Marshall et al., 2005). We expressed PKD2–NG in *fla10* cells, which harbor a fast-acting temperature-sensitive allele of a subunit of the anterograde IFT motor heterotrimeric kinesin-2, which can be used to turn off IFT via a temperature shift (Kozminski et al., 1995; Walther et al., 1994). After incubating *fla10 pkd2 PKD2-NG* cells for 80 min at the restrictive temperature of 32°C, cilia had shortened to an average length of 8.2 μm, compared to 9.5 µm of *fla10 pkd2 PKD2-NG* cells maintained at 22°C (Fig. 2A–C,Df,g, Table 1). During temperature-induced cilia shorting, the length ratio between the distal and proximal region remained at ∼2, similar to the observations during NaPPi-induced cilia shortening. In the *fla10* mutant at 32°C and NaPPi-treated cells, both regions shortened largely proportionally to the overall decrease in the cilia length and the position of the proximal border of the distal PKD2–NG region moved closer to the ciliary base (Fig. S2B). In the *fla10 pkd2 PKD2-NG* strain, the principal organization of PKD2 in a distal and a proximal region and the ratio between the two regions were maintained at the restrictive temperature, indicating that active IFT is not required to maintain or alter the length of the PKD2 regions.

The two rows of mastigoneme-associated PKD2 in the distal ciliary region are oriented roughly perpendicular to the plane of ciliary beating, which raised the possibility that ciliary motility was contributing to the organization of PKD2–NG (Liu et al., 2020). However, in a *pf19 pkd2 PKD2-NG* strain, which has paralyzed cilia due to the lack of the central pair apparatus, the organization of PKD2–NG in cilia was maintained, casting doubt on this hypothesis (Fig. 2A–C,Dh,i, Table 1) (Dymek and Smith, 2012).

Fig. 2E provides an overview of the relationship between ciliary length and the length of the two PKD2–NG regions in the various strains and conditions. In TIRFM images, the visible length of full-length control cilia is typically around 10 µm and accordingly, the data were separated into cilia below or above 10 µm length to distinguish regenerating, shortening and full-length cilia from and anomalously long cilia. The distal segment increases almost linear with ciliary length (∼0.8 µm/µm of cilia length) and continues to increase in length in abnormally long cilia. In contrast, the proximal region increases in length at 0.25 µm/µm in cilia shorter than 10 µm length; this rate decreased to just 0.12 µm/µm in cilia exceeding 10 µm in length. To summarize, the distal and proximal PKD2 region adjust in length when cilia grow or shorten; however, in abnormally long cilia, it is primarily the distal PKD2–NG region that increases in size (Fig. 2E).

### Efficient assembly of PKD2–NG into the distal region requires *de novo* assembly of cilia

**Fig. 3.**
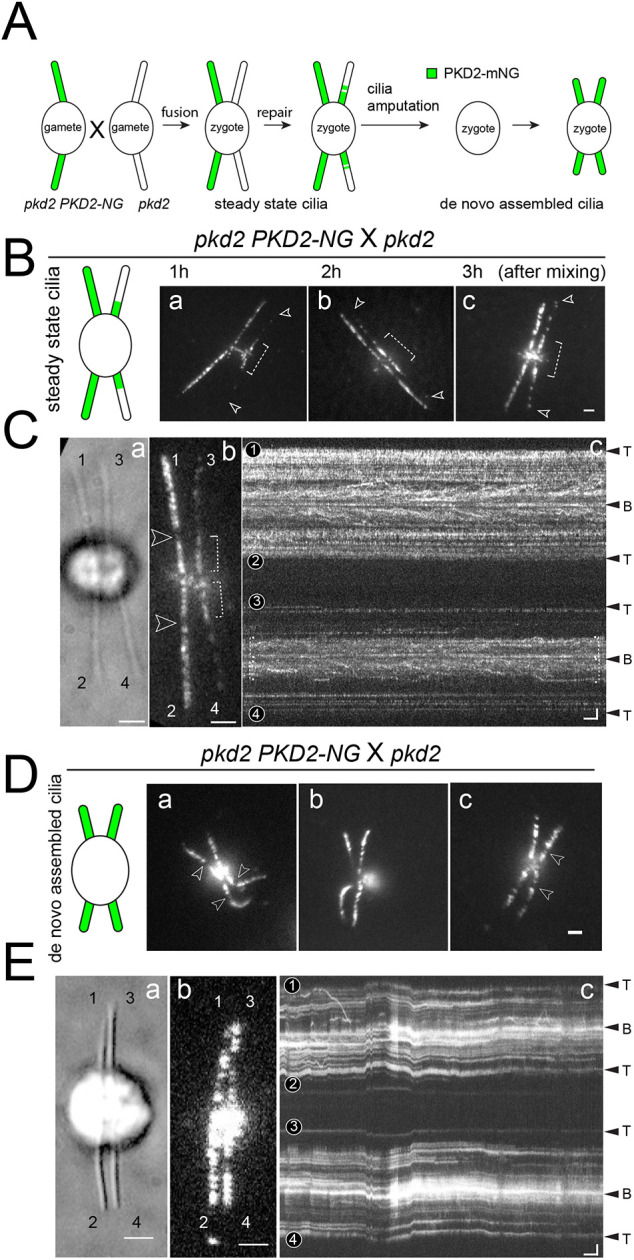
**Assembly of PKD2 into the distal region of pkd2 cilia requires *de novo* cilia.** (A) Schematic representation of a *Chlamydomonas* mating experiment followed by cilia amputation from the zygote and regeneration. (B) Schematic and TIRF images of *pkd2 PKD2-NG*×*pkd2* zygotes*.* Images were recorded at ∼1 h (a), 2 h (b), and 3 h (c) after mixing of the gametes. Arrowheads indicate the tips of the *pkd2* mutant-derived cilia. The proximal regions are marked with dotted brackets. Scale bar: 2 µm. (C) Bright field (a), TIRF image (b) and corresponding kymogram (c) of a *pkd2 PKD2-NG*×*pkd2* zygote with full-length cilia; the zygote was imaged ∼50 min after mixing of the gametes. Cilia derived from the *pkd2 PKD2-NG* parent are labeled 1 and 2; those derived from the *pkd2* mutant are labeled 3 and 4. The gaps (arrowheads), proximal regions (dotted brackets), and ciliary tips (T) and bases (B) are marked. Scale bars: 2 µm (a,b), and 2 µm and 2 s (c). (D) Schematic and TIRF images of *pkd2 PKD2-NG*×*pkd2* zygotes at ∼60 min after deciliation of the cells in the mating mixture by a pH shock. Arrowheads mark the gap between the two PKD2-NG regions. Scale bar: 2 µm. (E) Bright field (a), TIRF image (b) and corresponding kymogram (c) of a *pkd2 PKD2-NG*×*pkd2* zygote recorded at ∼40 min after the pH shock. The cilia are labeled 1 to 4; the ciliary tips and bases are marked. Scale bars: 2 µm (a,b), and 2 µm and 2 s (c). Images are representative of 44 (B,C) and 39 (D,E) zygotes analyzed.

To determine whether PKD2 can be added in the correct pattern to fully assembled *pkd2* mutant cilia, we mated *pkd2* and *pkd2 PKD2-NG* gametes (Fig. 3). After cell fusion, PKD2–NG present in the shared cytoplasm of the zygotes, is available for incorporation into the *pkd2*-derived cilia, which initially lack PKD2 (Fig. 3A). In zygotes analyzed ∼1 h after mixing of the gametes, PKD2–NG had entered the proximal one-third of the *pkd2-*derived cilia but only a very few particles were present in the distal region (Fig. 3B,C). An incomplete rescue of the *pkd2*-derived cilia was also observed in zygotes analyzed 2 or 3 h after mixing of the gametes with PKD2-NG largely restricted to the proximal region in those cilia; the distribution of PKD2–NG in the *pkd2 PKD2-NG*-derived cilia remained apparently unaltered (Fig. 3B,C). Such incompletely rescued zygotes accounted for 88% of the zygotes summarized from all three time points with the remaining 12% of zygotes having an overall weak or no detectable PKD2–NG signal in the cilia (*n*=44 zygotes analyzed). The latter can be attributed to low PKD2–NG expression in a subset of cells, as is frequently observed with transgenes in clonal cultures of *Chlamydomonas*.

To test whether the lack of PKD2 assembly into the distal region is a zygote-specific feature, we deciliated zygotes using a pH shock and allowed them to regenerate all four cilia (Fig. 3A,D,E). In 77% of the 39 zygotes analyzed, the cilia displayed the normal compartmentalization of PKD2–NG with a 1:2 length ratio between the proximal and distal region (Fig. 3D,E). The remaining zygotes either largely lacked PKD2–NG or, more frequently, possessed two incompletely rescued and two normal cilia; the latter zygotes were likely derived from gametes present in the mating mixture at the time of the pH shock and that only fused after deciliation and cilia regeneration (data not shown).

We also mated *pkd2 PKD2-NG* and wild-type gametes to visualize the exchange of untagged PKD2 in the wild-type-derived cilia with PKD2–NG (Fig. S2B). As described above, PKD2–NG quickly entered the proximal one-third of the two wild-type-derived cilia in 91% of zygotes analyzed but was largely excluded from the distal region of those cilia. These data indicate that PKD2–NG assembly into full-length zygotic cilia is largely limited to the proximal mobile region, in which PKD2 is more dynamic and in exchange with PKD2 in the cell body. Further, anchoring of PKD2 complexes to the axoneme appears to occur preferentially during ciliary assembly.

### Identification of SIP as a novel PKD2-associated protein

We hypothesized that the complex arrangement of PKD2 observed in *Chlamydomonas* cilia likely involves additional proteins. To identify additional components of the ciliary PKD2–MST1 complex, we immunopurified PKD2–NG from detergent extracts of *pkd2 PKD2-NG* and *mst1-1 pkd2 PKD2-NG* cilia using an anti-NG nanobody trap (Fig. S3A). The latter strain was chosen because the lack of MST1 reduces the presence of PKD2–NG in the distal cilia region (Fig. S3B) (Liu et al., 2020). Therefore, the levels of proteins specifically interacting with PKD2 in the distal region could be also reduced in *mst1-1* cilia in comparison to those of the *pkd2 PKD2-NG* rescue strain. The wild-type strain g1 was used as a control. Silver staining identified several bands in the eluates of the PKD2–NG-expressing strains that were absent in the control eluates (Fig. S3A). A prominent band of ∼250 kDa was present in the *pkd2 PKD2-NG* eluate but not in that of the *mst1-1 pkd2 PKD2-NG* and control strains, and likely represents MST1. The analysis was carried out in several biological replicates (four for the *pkd2 PKD2-NG* and three for the *mst1-1 pkd2 PKD2-NG* and the control strain, respectively) and the eluates were subjected to mass spectrometry (Table S1). Certain abundant (e.g. tubulin) and ‘sticky’ proteins (e.g. FMG-1B) were detected in all ten samples; PKD2 was detected in the seven experimental samples but not in the controls. As expected, MST1 was only present in the samples from the *pkd2 PKD2-NG* rescue strain. An additional 27 proteins were identified in most of the *pkd2 PKD2-NG* and/or *mst1-1 pkd2 PKD2-NG* samples (Table S1). Although only present in the experimental samples, we noticed that this list also encompassed proteins that we repeatedly detected in GFP and NG pulldowns in unrelated experiments (e.g. ODA5-associated adenylate kinase and enolase) and proteins, such as chlorophyll-binding proteins, which are cell body contaminants.

**Fig. 4.**
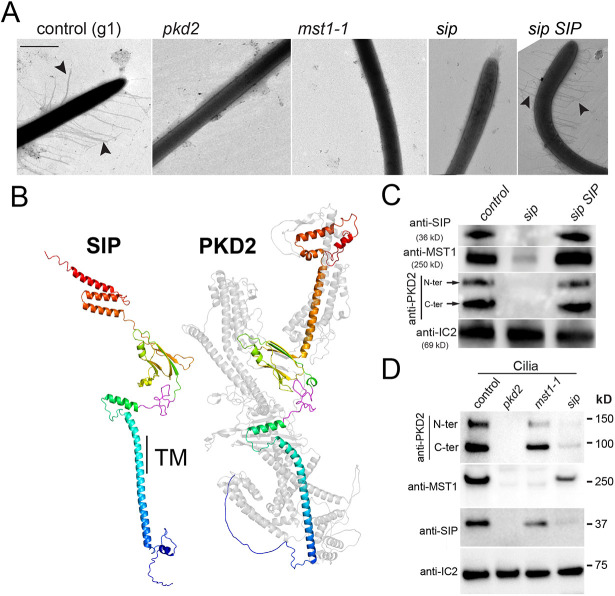
**Ciliary presence of the PKD2–mastigoneme complex requires SIP.** (A) Analysis of cilia from control, *pkd2*, *mst1-1*, *sip* mutant and *sip SIP* rescue cells by whole-mount EM. Arrowheads, mastigonemes. Images are representative of 30 or more cells analyzed. Scale bar: 600 nm. (B) Alphafold2 comparison of SIP and PKD2. For PKD2, only the parts corresponding to SIP are shown in color. The colors indicate the orientation of the N-terminal (blue) and more C-terminal (red) regions. Magenta indicates the 30-residue stretch that is well conserved between SIP and PKD2 (see Fig. S4C). TM, position of the transmembrane domain of SIP. (C) Western blot analysis of cilia isolated from control, the *sip* mutant and the *sip SIP* rescue strain. The membrane was probed with anti-SIP, anti-MST1 and anti-PKD2 antibodies and, as a control for equal loading, anti-IC2 antibody, which labels a subunit of the outer dynein arms. Ciliary PKD2 runs as two bands of ∼90 kDa (representing the C-terminal fragment) and ∼140 kD (representing the N-terminal fragment). (D) Western blot analysis of isolated cilia from control and the *pkd2*, *mst1-1* and *sip* mutant cells probed with anti-PKD2, anti-MST1, anti-SIP and anti-IC2 antibodies. Note traces of SIP are present in the *sip* mutant, indicating that some expression of the protein was restored. The western blots are representative of three (C) and four (D) biological repeats.

To triage candidate interacting proteins, we obtained known or putative mutants in genes encoding proteins specific for the experimental samples and processed them for whole-mount EM (Table S1). Of the six strains analyzed, only strain LMJ.RY0402.143879 from the *Chlamydomonas* CLiP mutant collection lacked mastigonemes (Fig. 4A). The absence of mastigonemes was confirmed by immunofluorescence staining with monoclonal anti-MST1 antibody (Nakamura et al., 1996), which further revealed that the pool of MST1/mastigonemes observed in the apical region of control cells, is dispersed in LMJ.RY0402.143879 cells, as previously described for the *pkd2* mutant (Fig. S4A) (Liu et al., 2020; Nakamura et al., 1996). This strain carries an insertion on chromosome 11 in the second intron of CHLRE_11g475150, which encodes the uncharacterized protein A8JFQ9_CHLRE (Fig. S3C). The protein is predicted to consist of 361 residues and to possess a single transmembrane domain. It is annotated as ‘similar to PKD2’ in the Phytozome database (https://phytozome-next.jgi.doe.gov/) because it shares similarity with the N-terminal region of *Chlamydomonas* PKD2, including a stretch of 30 residues with 80% identity (Fig. S4B,C and Fig. 4B, depicted in magenta). Alphafold2 predicts remarkably similar structures for A8JFQ9_CHLRE and the N-terminal region of *Chlamydomonas* PKD2, encompassing the first transmembrane helix and parts of the extracellular top domain (Fig. 4B). In NCBI Blastp searches of *Chlamydomonas* proteins, PKD2 and A8JFQ9_CHLRE were reciprocal second-best hits for each other (E value 7×10^−30^). For reasons of simplicity, we will refer to A8JFQ9_CHLRE as Small Interactor of PKD2 (SIP). SIP was the only protein enriched in the pulldowns of the *pkd2 PKD2-NG* strain compared to the *mst1-1* sample (Table S1). The *SIP* gene is present in the genomes of various green alga (i.e. Chlorophyta) including Chlamydomodales with MST1-based mastigonemes, such as *Volvox carteri*, as well as species without MST1, such as *Trebouxia* sp. and *Micromonas* sp., and species that apparently lack the ability to form cilia (e.g. *Scenedesmus* sp.). Outside of green algae, homologs of *Chlamydomonas* SIP were not detected (Table S2).

A polyclonal antibody raised against recombinant SIP identified a band of ∼36 kDa in western blots of isolated control cilia, which is close to the predicted molecular mass of SIP of 39,718 (Fig. 4C; Fig. S4D). The immunoreactive band was absent in cilia from strain LMJ.RY0402.143879, revealing that this mutant lacks SIP; we therefore refer to this strain as *sip* (Fig. 4C,D; Fig. S4B). PKD2 and MST1 were largely absent from *sip* mutant cilia (Fig. 4C). For rescue, we expressed untagged SIP in the *sip* mutant; the presence of both the transgenic cDNA-based and the insertional mutant alleles was confirmed by PCR (Fig. S3D,E). Western blotting showed that expression of SIP restored PKD2 and MST1 levels in cilia, and whole-mount EM showed the presence of mastigonemes on *sip SIP* cilia (Fig. 4A,C). Of note, during the course of this study, the *sip* mutant occasionally regained the ability to express some SIP (Fig. 4D). Although not further analyzed here, it is likely that cells occasionally acquired the ability to splice out the large intron generated by the insertion of the selectable marker cassette into intron 2 (Fig. S3C). Cilia of control and *sip* mutants reacted equally with anti-SIP in immunofluorescence assays, suggesting that this antibody is not suitable for immunocytochemistry. We also failed to express tagged SIP in *Chlamydomonas*; therefore, the localization of SIP within cilia remains unknown. However, we note PKD2 and SIP co-fractionated during Triton X-114 phase partitioning of isolated cilia and were mostly present in the soluble matrix fraction with a minor portion remaining attached to the axonemes (Fig. S4E).

To further analyze the interdependence between PKD2, SIP and mastigonemes, cilia were isolated from control cells and the corresponding mutants, and then compared by western blotting using antibodies directed against PKD2, SIP and MST1 (Fig. 4D). In our hands, monoclonal anti-MST1 failed to detect MST1 in western blots and, hence, we raised a novel polyclonal antibody against a 174-residue fragment of MST1 encoded by exon 14 (i.e. residues 1307–1480); this polyclonal anti-MST1 identified MST1 in western blot experiments but was not suitable for immunofluorescence approaches (Fig. S4F). As expected, all three proteins were detected in control cilia with PKD2 running as two bands, the larger N-terminal fragment and the smaller C-terminal fragment, as previously reported (Fig. 4C,D) (Huang et al., 2007; Liu et al., 2020). In the *pkd2* mutant, MST1 and SIP were not detected, indicating that PKD2 holds a central role in the complex and is required for the ciliary presence of MST1 and SIP (Fig. 4D). In *mst1-1* cilia, PKD2 and SIP were present but their levels were significantly reduced (Fig. 4D). PKD2 and MST1 were strongly reduced (Fig. 4D) or undetectable (Fig. 4C) in *sip* mutant cilia, revealing that SIP is required for the presence of PKD2–mastigoneme complexes in cilia.

**Fig. 5.**
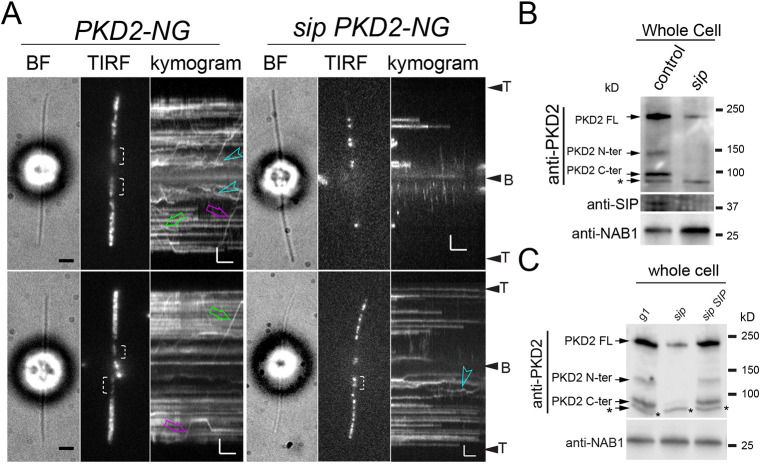
**SIP is required for proteolytic cleavage of PKD2.** (A) Bright field (BF) and TIRF images and the corresponding kymograms of *PKD2-NG* and *sip PKD2-NG* cells. Discernable proximal PKD2 regions are marked by brackets; green and magenta arrows indicate anterograde and retrograde IFT; blue arrowheads mark PKD2–NG diffusion. The ciliary tips (T) and bases (B) are indicated. Scale bars: 2 µm and 2 s. Images are representative of 25 or more cells analyzed. (B) Western blot analysis of whole-cell samples of the control and *sip* strain with anti-PKD2 and anti-SIP antibodies, and for NAB1, a nuclear protein that serves as a loading control for whole-cell samples. Note the reduction of full-length PKD2 (FL) and near absence of the N- and C-terminal fragments of PKD2 in the *sip* mutant. (C) Western blot of whole-cell samples of control (g1), the *sip* mutant, and the *sip SIP* rescue strain probed with anti-PKD2 antibody and anti-NAB1 antibody, as a loading control. Full-length PKD2 and the fragments are indicated. Note the near absence of PKD2 fragments in *sip* mutant strains. *, cross-reacting unspecific bands. The western blots are representative of three (B) and five (C) biological repeats.

To analyze the behavior of residual PKD2 in *sip* cilia, we expressed PKD2–NG in the *sip* mutant and a control strain, which both also expressed the endogenous PKD2 (Fig. S4G). As expected from the biochemical analysis of cilia (Fig. 4C,D), PKD2–NG was severely reduced (∼10% of cells analyzed) or not detected (∼90%) in *sip* cilia (Fig. 5A). Residual PKD2–NG was mostly stationary. Furthermore, an organized pool of PKD2–NG with the protein present near the basal bodies and along the microtubular cytoskeleton seen in control cells, was not observed in *sip PKD2-NG* cells (Fig. S1A,B).

The near absence of PKD2 from *sip* mutant cilia raised the possibility that PKD2 is trapped in the cell body of these mutants. In immunoblots loaded with control whole-cell samples, anti-PKD2 recognized full-length PKD2 (∼230 kDa) and the two proteolytic fragments of 90 and 140 kDa, corresponding to the C- and N-terminal fragments of PKD2 (Huang et al., 2007; Liu et al., 2020) (Figs 4C,D and 5B,C). In *sip* cells, the overall amount of PKD2 was reduced and, interestingly, residual PKD2 was mostly uncleaved whereas the proteolytic fragments were essentially undetectable (Fig. 5B,C). Expression of SIP in *sip* mutants was able to rescue normal levels of both PKD2 and the PKD2 fragments (Fig. 5C). Huang et al. (2007) observed proteolytic cleavage of PKD2 in the cilia-deficient *Chlamydomonas* mutants *bld1* and *bld2*, indicating that cleavage occurs in the cell body and that only the two fragments enter the cilia (Huang et al., 2007). We conclude that SIP is required for the stability and proteolytic processing of PKD2 in the cell body, the latter being a likely prerequisite for the entry of PKD2 into *Chlamydomonas* cilia.

### The PKD2–mastigoneme complex increases the efficiency of the ciliary beat

**Fig. 6.**
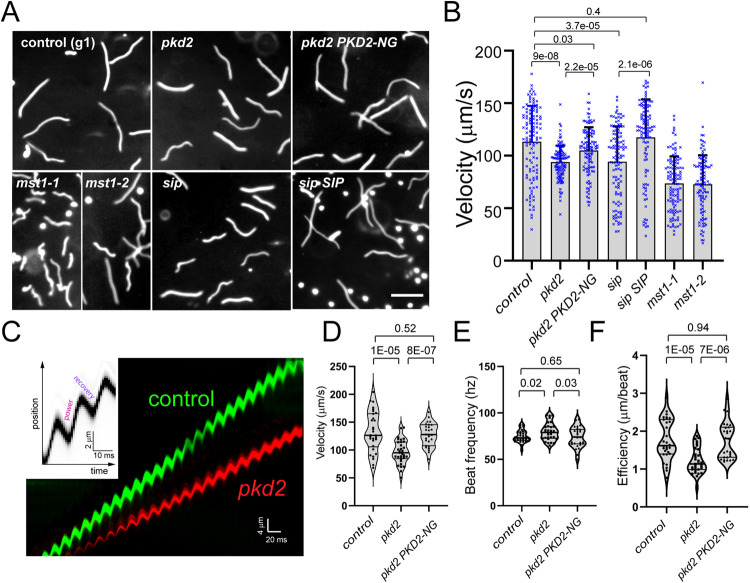
**Loss of PKD2–mastigoneme complexes reduces the efficiency of the ciliary beat.** (A) Micrographs obtained at 1-s exposures showing the swimming paths of control, the *pkd2*, *mst1-1, mst1-2* and *sip1* mutants, and the *pkd2 PKD2-NG* and the *sip SIP* rescue strains. Scale bar: 100 µm. (B) Bar graph showing the swimming velocity of various strains as determined by the ‘LengthAnalysisTool’ (see Materials and Methods). The standard deviations, individual data points and results of two-tailed unpaired *t*-tests are indicated. (C) Overlay kymogram based on high-speed recordings of a control (g1; green) and a *pkd2* mutant (red) cell. The insert marks the power and recovery stroke in a kymogram. Note that both cells have a similar beat frequency but the *pkd2* cell moves with reduced velocity. (D–F) Violin plots of the velocity (D), ciliary beat frequency (E) and beat efficiency (F) of control, *pkd2* and *pkd2 PKD2-NG* cells. The results of two-tailed unpaired *t*-tests are indicated. Shown are the data from one of two experiments with similar outcomes, each involving 20 or more cells per strain.

The swimming velocity of the *sip* mutant was reduced by ∼20%, similar to that of the *pkd2* and *mst1-1* mutants (Fig. 6A,B,D). Expression of transgenic SIP in the *sip* mutant rescued the motility phenotype. Here, we also analyzed *mst1-2* (also known as *mstg*, which is CLiP strain LMJ.RY0402.136134; Table S3), a strain that lacks mastigonemes and was previously shown to swim with normal velocity (Amador et al., 2020). In our hands, when applying our semi-automated analysis of swimming trajectories, *mst1-2* instead swam with reduced velocity (Fig. 6B; Fig. S5). To further analyze how PKD2 and its associated proteins MST1 and SIP promote fast swimming of *Chlamydomonas*, high speed video recordings were analyzed by visual examination and kymography (Fig. 6C). The beat frequency of the *pkd2* mutant cilia was slightly elevated, but we observed that the beat efficiency (i.e. the distances a cell moves during each beat cycle) was greatly reduced compared to those of control and *pkd2 PKD2-NG* rescue cells, providing a likely explanation for the reduced swimming velocity of the *pkd2* mutant (Fig. 6E,F). This observation supports a role of the PKD2–mastigoneme complex in increasing the effective surface of the cilia, allowing for faster swimming. This concept could also explain the somewhat increased beat frequency of *pkd2* mutant cilia in comparison to the wild-type and rescue strains, as the absence of mastigonemes will likely reduce the resistance experienced by the beating cilia (Fig. 6E).

## DISCUSSION

Here, we analyzed the assembly of PKD2 in *Chlamydomonas* cilia, building on our previous observation that PKD2–NG is subcompartmentalized along the proximo-distal axis of cilia. Using *Chlamydomonas*, we addressed three questions. (1) How do the PKD2 regions develop and adjust in response to changing parameters such as cilia length? (2) How does the lack of PKD2 affect ciliary motility? (3) Does the formation of PKD2 patterns involve additional proteins?

### Is the proximal PKD2 region a ciliary sorting compartment?

In *Chlamydomonas* cilia, two populations of PKD2–NG can be distinguished: PKD2–NG in the distal region is immobile, has a low turnover and binds mastigonemes, whereas the proximal region lacks mastigonemes and PKD2–NG is more mobile and quickly exchanges with PKD2 in the cell body. The proximal region neighbors the transition zone and all ciliary PKD2, with and without mastigonemes, will pass into this region when entering cilia. The complex tripartite mastigonemes of the heterokont *Ochromonas* are present in secretory in vesicles with their base already anchored to the vesicular membrane and remaining membrane-anchored during secretion near the ciliary base (Bouck, 1971). In *Chlamydomonas*, loss of PKD2 (or SIP) affects the accumulation of MST1 near the ciliary base, suggesting that all three proteins move as a complex to secretion sites near the cell apex (Liu et al., 2020). Mastigonemes were not observed in the proximal region of cilia, including within short regrowing cilia, suggesting that after entering the cilium, mastigoneme–PKD2 complexes quickly pass into the distal region (Liu et al., 2020). In contrast, mastigoneme-deficient PKD2–NG complexes in the *mst1-1* mutant are largely contained within the proximal region rather than dispersing along the cilia. Thus, the proximal ciliary region could function as sorting compartment permitting PKD2–mastigoneme complexes to quickly pass into the distal cilium for anchoring while retaining mastigoneme-deficient PKD2. This leaves open the question of the mechanism by which the margins of the PKD2 regions are defined, particularly the distal border of the proximal region. One possibility is the presence of weaker transient binding sites for PKD2–NG in the proximal region and more stable, PKD2–mastigoneme-specific docking sites on DMTs 4 and 8 in the distal region; the gap could be explained by an intercalated region without PKD2-binding sites. Alternatively, the distal border of the proximal region could contain a gate, fencing in PKD2 without mastigonemes, while still permitting PKD2–mastigoneme complexes to pass into the distal cilium. Although the transition zone is the main ciliary gate (Garcia-Gonzalo and Reiter, 2012), additional diffusion barriers within the ciliary membrane cannot be excluded. Indeed, Lee et al. reported partitioning of the ciliary membrane along the length of cilia into actin-dependent corals, transiently confining diffusing G-protein coupled receptors (Lee et al., 2018). Furthermore, the ciliary dilation of chordotonal neuron cilia in *Drosophila* defines or maintains the border between the proximal and distal zone (Xiang et al., 2022) and, in sperm flagella, the septin-based annulus forms a diffusion barrier between the midpiece and principal piece (Kwitny et al., 2010).

The presence of a distinct PKD2–NG region in the proximal cilium of *Chlamydomonas* is only one of several known structural, biochemical and functional specializations of this region. Ciliary bending is initiated in the proximal region, which possesses a special subset of inner dynein arms of unknown function (Yagi et al., 2009) and a bridge between DMTs 1 and 2. Also, the dynein assembly factor ODA10 is specific for the proximal ∼3–4 µm of cilia (Dean and Mitchell, 2015), and the kinases FA2 and LF5 are restricted to the very proximal end of the ciliary shaft, just above the transition zone (Mahjoub et al., 2002; Tam et al., 2013). Similarly, primary cilia often possess a peri-axonemal ‘inversin compartment’ in the proximal region, which is crucial for proper ciliary signaling during left–right asymmetry determination and in kidney development (Bennett et al., 2020; Mochizuki et al., 1998; Shiba et al., 2009). Our data suggest that the proximal region of cilia might be a sorting compartment, ensuring that only fully assembled PKD2–SIP–MST1 complexes enter the more distal cilium whereas mastigoneme-deficient PKD2 complexes are retained.

### The length of PKD2 regions is adjusted in a ciliary length-dependent manner

The plus-ends of the axonemal microtubules point toward the ciliary tip, and axonemes grow by addition of tubulin to the distal plus-end, with other axonemal substructures added briefly after (Euteneuer and McIntosh, 1981; Hoog et al., 2014; Lechtreck et al., 2013; Rosenbaum and Child, 1967; Witman, 1975). In a simple model, proteins specific for the proximal region of full-length cilia are delivered and assembled early during cilia formation, whereas those specific for more distal regions will follow later. However, our data reveal that the PKD2 regions are not established sequentially as cilia grow. Rather, the two PKD2 regions develop early during ciliogenesis and proportionally adjust in length as cilia elongate. This implies that PKD2 is redistributed, for example, the proximal border of the distal region must be moved distally as cilia grow, a process that likely involves changes in the underlying axoneme to generate or eliminate PKD2-binding sites. During the assembly of *Drosophila* auditory cilia, dynein motor complexes are initially not confined to their proximal target zone, and ectopic complexes initially observed in the distal zone, are later removed (Xiang et al., 2022). Also, the OcNC1 channel subunit, initially present in the proximal segment of assembling rat olfactory cilia, is later moved to its final position in the distal segment (Matsuzaki et al., 1999). Cilia maturation seemingly involves the trimming and rearranging of proteins incorporated during earlier stages of assembly. For *Chlamydomonas* PKD2, such dynamics also occur when cilia shorten, given that the proportionality of the PKD2-NG regions is essentially maintained in shortened cilia. This indicates that ciliary resorption is not a simple breakdown from the tip but, at least with respect to *Chlamydomonas* PKD2, involves reorganization and rescaling.

### Efficient axonemal docking of PKD2 requires *de novo* assembly of cilia

In the distal region of cilia, PKD2–mastigoneme complexes are anchored to just two of the nine doublets, suggesting that only these two doublets provide accessible docking sites for the complex (Liu et al., 2020). Similarly, ODA10 is targeted with high precision to the proximal region of DMT 1 (Dean and Mitchell, 2015). Furthermore, numerous structural specializations typical for one or a subset of the nine DMTs, for example, the B-tubule beaks, DMT 1-to-2 bridge, absence of ODAs from DTM 1 etc., have been identified in *Chlamydomonas* cilia, indicating the presence of biochemical differences between the different doublets (Bui et al., 2012; Dutcher, 2020; Hoops and Witman, 1983). The DMTs are continuous with the basal body triplets, each of which possess unique features with respect to their association to basal apparatus fibers and ciliary roots, centrin fibers within the triplet cylinder, and their position within the cell and with respect to the mother basal bodies during their genesis (Geimer and Melkonian, 2004; Holmes and Dutcher, 1989; Wingfield and Lechtreck, 2018). Likely, distinct features of the triplet microtubules determine the individual characteristics hardwired into the axonemal DMTs. PKD2–NG docking into the distal region of fully assembled cilia is a rather slow process. Perhaps, the docking sites on the DMTs 4 and 8 are obscured or absent in such full-length zygotic cilia and efficient axonemal anchoring of PKD2 into two rows requires *de novo* assembly of cilia. Possibly, parts of PKD2 or a putative linker co-assembles during DMT elongation, whereas appending the connection to fully formed DMTs could be difficult.

### The PKD2–SIP–mastigoneme complex increases the efficiency of the ciliary beat

Previously, we have shown that the *pkd2* and *mst1-1* mutants swim with moderately (reduced by ∼20%) reduced velocity (Liu et al., 2020). Similarly, Nakamura et al. (1996) observed a 20–30% reduction of the swimming velocity combined with a slight (∼10%) increase in beat frequency after mastigonemes were removed from control cilia by treatment with a monoclonal antibody against MST1 (Nakamura et al., 1996). In a recent study by Wang and colleagues, only a slight reduction in swimming velocity was reported for *mst1-1*; the mutant, however, swam slower than controls at high viscosity and displayed reduced gravitaxis (Wang et al., 2023). Swimming velocity was unaffected in *mst1-2*, another CLiP strain lacking mastigonemes (Amador et al., 2020; Wang et al., 2023). In *Chlamydomonas*, swimming velocity varies greatly depending on culture conditions, cell density, time of the day and assay conditions, such as light and temperature, and a standardized approach to determine swimming speed is missing. Here, we developed a simple plugin for Fiji/ImageJ to extract swimming velocities from long-exposure micrographs, allowing us to analyze a large number of cells. Using this tool, we determined that the *pkd2*, *sip* and both *mst1* mutants all swam with reduced velocity. A common feature of all four mutants (i.e. *pkd2*, *sip*, *mst1-1* and *mst1-2*) is the absence of mastigonemes from cilia. These ∼800-nm long hairs project from both sides of the cilium and are oriented approximately perpendicular to the plane of ciliary beating. Assuming that the mastigonemes are sufficiently stiff to somewhat project laterally from cilia rather than being dragged behind, they would increase the surface area of the beating cilium. This would be expected to improve the efficiency of the ciliary beat, allowing cells to travel a larger distance during each beat cycle compared to cells without mastigonemes. High-speed video revealed that *pkd2* cells traveled less than control cells during each ciliary beat cycle, while the bending motion was similar, and the beat frequency was slightly (∼8%) increased. The latter value is similar to what was found upon observation of control cells after experimental removal of the mastigonemes (Nakamura et al., 1996). Compared to controls, the beat frequency was also higher for *mst1-2* but the difference was not significant (Amador et al., 2020). These data support a parsimonious model for the function of *Chlamydomonas* PKD2 in which its prime role is to anchor the mastigonemes to the ciliary surface, forming a fan-like superstructure, which increases the ciliary surface, beat efficiency and swimming velocity. However, this concept generates a conundrum because the overall domain structure and sequence of *Chlamydomonas* and mammalian PKD2 is well conserved, suggesting that *Chlamydomonas* PKD2 is a functional channel (Huang et al., 2007). The phenotypical defects of *Chlamydomonas pkd2* described so far are mild but this does not exclude a role for PKD2-based ion currents in *Chlamydomonas* cilia during behaviors that are difficult to recognize and assay. As PKD2 is reduced in *mst1* cilia and *pkd2* cilia lack mastigonemes, assigning an individual role to each protein is difficult. Loss-of-function and gain-of-function PKD2 mutants, which still bind mastigonemes and the axoneme, could allow for a better assessment of the putative channel function of *Chlamydomonas* PKD2.

### SIP promotes proteolytic processing and ciliary entry of PKD2

To identify proteins required for ciliary targeting and patterning of PKD2, we isolated PKD2 complexes from ciliary detergent extracts and identified the single-pass transmembrane protein SIP as an interactor of *Chlamydomonas* PKD2. Mammalian PKD2 interacts with the triple-pass transmembrane protein TMEM33 in the ER and the 11-transmembrane domain protein PKD1 in cilia; single-pass interactors of PKD2 were not identified (Arhatte et al., 2019; Wu and Somlo, 2000). Single-pass transmembrane proteins, however, are part of the ciliary CatSper channel complex in mammalian sperm flagellar. This complex contains at least three single-pass transmembrane proteins (CatSper γ, CatSper δ and CatSper ζ), which are required for proper trafficking, assembly and/or function of the complex in the principal piece of sperm flagella (Chung et al., 2017; Singh and Rajender, 2015). Furthermore, Na^+^ channels encompass single-pass β-subunits, which regulate channel gating, localization and anchoring to the cytoskeleton (Isom, 2001). Interestingly, SIP is highly reminiscent of the N-terminal region of *Chlamydomonas* PKD2, giving it the appearance of a PKD2 fragment. In detail, SIP corresponds to the non-pore-forming transmembrane helix 1 and parts of the extracellular top domain of PKD2. Our data indicate that *Chlamydomonas* SIP is required for the processing of PKD2 in the cell body, before traveling together with PKD2 into cilia. In *Chlamydomonas*, PKD2 is cleaved within its large extracellular domain between helix 1 and 2 and only the two resulting fragments, which remain associated, enter cilia (Huang et al., 2007; Liu et al., 2020). In *sip* mutants, the overall amount of PKD2 is reduced, the level of its fragments was severely diminished, and its apical accumulation was not apparent. We propose that proteolytic processing of PKD2 is a prerequisite for its entry into cilia and that SIP somehow participates in PKD2 processing, for example, by contributing to the assembly of a cleavable PKD2 complex, ensuring its proper localization, or by generating a site on the complex to recruit a protease (Fig. S4G). In mammals and *C. elegans*, polycystin 1 (PKD1), the binding partner of PKD2 in these organisms, undergoes cleavage at the G-protein-coupled receptor proteolysis site motif in the extracellular domain; proteolytic processing of PKD1 is involved in its localization to the cell surface and, in *C. elegans*, is relevant for its localization to cilia (Chapin et al., 2010; Walsh et al., 2022). To summarize, our data suggest a mutual co-dependency of PKD2, SIP and MST1/mastigonemes for entry into *Chlamydomonas* cilia.

The presence of SIP-encoding genes is limited to the genomes of various green algae, including those with *Chlamydomonas*-like mastigonemes, but also other species that lack mastigonemes/MST1 and even cilia. This suggests that SIP is a green algal PKD2-interacting protein rather than being specifically required for the binding of PKD2 to mastigonemes and DMTs. Axonemal docking of PKD2, however, might involve a component, which is tightly associated with DTMs 4 and 8 and therefore not released by detergent treatment as used here to identify SIP. Future work using proximity labeling techniques; i.e., via expression of PKD2- or SIP-biotin ligase fusions, might provide a strategy to shed light on axonemal docking of PKD2.

## MATERIALS AND METHODS

### Strains, culture conditions and genotyping

*Chlamydomonas* strains used in this study are listed in Table S3. The wild-type strain CC-620, the mutant strains *lf4* (CC-4534) and *fla10* (CC-1919), and the rescue strain *pkd2 PKD2-NG* (CC-5899) are available from the Chlamydomonas Resource Center. The original *pkd2* mutant (LMJ.RY0402.204581), and the *sip* (LMJ.RY0402.143879) and *mst1-1* (LMJ.RY0402.052413) and *mst1-2* (LMJ.RY0402.136134) strains were obtained from the *Chlamydomonas* Library Project (https://www.chlamylibrary.org/allMutants; Li et al., 2019). CC-5235 and g1 (Pazour et al., 1995) were used as wild-type controls. The *mst1-1 pkd2 PKD2-NG*, *lf4 pkd2 PKD2-GFP*, *fla10 pkd2 PKD2-GFP* were generated by mating. The *pkd2 PKD2-GFP* was as previously described (Liu et al., 2020). Here, we used a *pkd2* mutant that was outcrossed twice with g1. Cells were grown in modified minimal (M) medium (https://www.chlamycollection.org/methods/media-recipes/minimal-or-m-medium-and-derivatives-sager-granick/) and maintained at 22°C with a 14-h-light–10-h-dark cycle; large cultures used for cilia isolation were aerated with air enriched with 0.5% CO_2_.

### Transgenic strain generation

To rescue the *sip* mutant, cDNA of *SIP* gene was amplified from using primers 1 and 2 (Table S4) and *Chlamydomonas* cDNA as a template and inserted into pGenD (Fischer and Rochaix, 2001). To provide antibiotic resistance, the hygromycin cassette was amplified using primer 3 and 4 (Table S4) inserted into the pGenD-SIP plasmid. The resulting pGenD-SIP+Hyg plasmid was linearized using XbaI and transformed in the *sip* mutant by electroporation (Invitrogen Neon™ Transfection system). Transformants were selected on TAP plates containing 20 µg/ml hygromycin (Bio Basic). Clones expressing SIP protein were identified using whole-mount EM based on the restoration of mastigonemes on the ciliary surface, which was observed in one out of more than 60 transgenic clones analyzed. The presence of SIP, PKD2 and MST1 in cilia was confirmed by western blot analysis and the *sip SIP* genotype was confirmed using PCR using primers 5 and 6 (Table S4) to track the *sip* insertional allele and primers 7 and 2 to track the transgene; primers 8 and 9 were used to amplify a part of the g*β* gene to verify DNA quality (Zamora et al., 2004).

### Ciliary regeneration

Vegetative cells or zygotes in M medium were deciliated by a pH shock (pH 4.2 for 30 s), transferred to fresh M medium (nitrogen-free M medium for zygotes), and allowed to regrow cilia under constant light with agitation. Samples were analyzed at various time point during cilia regeneration by TIRFM.

### Mating experiments

To generate gametes, 100 ml of vegetative cells were grown for 4–5 days to a cell density of 2×10^6^ cells/ml. The evening prior to the mating experiment, cells were transferred to 15 ml nitrogen-free M medium and aerated overnight under constant light. In the morning, cells were transferred to 2 ml of 1/5th nitrogen-free M medium supplemented with 10 mM HEPES and incubated for an additional 30 min to 4 h, followed by mixing of gametes of opposite mating type. For TIRFM, the cell suspension was mounted for *in vivo* imaging at various time points after mixing of the gametes. The distribution of PKD2–NG in cilia was scored by visual examination. To obtain progeny, the cell suspension was incubated in light without agitation for 4–6 h, plated onto dry mating pates (TAP medium with 4% agar or 1.8% phytogel; https://www.chlamycollection.org/methods/media-recipes/tap-and-tris-minimal/), incubated overnight in light, air-dried and stored for >10 days in the dark. Plates were transferred to −20°C for 2 days, thawed, dried and incubated in constant light for several days. Colonies were streaked for single cells, and progeny with the desired traits was identified using TIRFM, geno- and pheno-typing, and PCR and western blotting.

### Isolation of cilia

To isolate cilia, cells were washed and concentrated in 10 mM HEPES (pH 7.4), resuspended in 10 ml of HEPES-Mg^2+^-sucrose (HMS; 10 mM HEPES, pH 7.4, 5 mM MgSO_4_, 4% sucrose), and immediately deciliated by adding 2.5 ml of dibucaine-HCl (25 mM in H_2_O; Sigma-Aldrich) and vigorous pipetting (Craige et al., 2013). After addition of 20 ml of 0.7 mM EGTA in HMS, the cell bodies were removed by centrifugation (1150 ***g***, 3 min, 4°C; Sorvall Legend XTR, Thermo Fisher Scientific). Next, the supernatant was underlaid with a sucrose cushion (10 ml of 25% sucrose in HMS) and the remaining cell bodies were removed by centrifugation (1700 ***g***, 4°C, 10 min). Cilia in the upper phase were sedimented by centrifugation (27,000 ***g***, 4°C, 20 min; Beckman Coulter, Avanti JXN-26), resuspended in HEPES -Mg^2+^-EGTA-K^+^ (HMEK; 30 mM HEPES, 5 mM MgSO_4_, 0.5 mM EGTA and 25 mM KCl) supplemented with 1% protease inhibitor cocktail (Sigma-Aldrich, P9599) and lyzed for 20 min on ice with Triton X-100 or, if phase partitioning was planned, Triton X-114 (each at 1% final concentration). The axonemes were separated from the membrane plus matrix fraction centrifugation (27,000× ***g***, 4°C, 15 min). For phase partitioning, the supernatant was incubated at 30°C for 5 min; phase separation is evident by the cloudy appearance of the solution. The micelles were harvested by centrifugation (1700 ***g***; 22°C; 5 min) leading to an upper aqueous phase (matrix fraction) and a detergent phase (membrane fraction). Proteins in the detergent phase were further concentrated by methanol-chloroform precipitation.

### Immunoprecipitation

Cilia isolated from *pkd2 PKD2-NG*, *mst1-1 pkd2 PKD2-NG* and an untransformed control strain were resuspended in HMEK supplemented with 100 mM NaCl (final concentration) and protease inhibitor cocktail (Sigma-Aldrich, P9599) and lysed by addition of 1% NP-40 (final concentration). The axonemes were removed by centrifugation (27,000 ***g***, 4°C, 15 min) and the supernatant was incubated with anti-NG nanobody agarose beads (Allele Biotechnology) for 1 h at 4°C using a rotisserie. The loaded beads were washed twice with HMEK containing 150 mM NaCl and bound proteins were eluted using 200 mM glycine, pH 2.5. The eluate, input and flow-through were analyzed using silver-stained gels (Silver Stain Plus Kit, Bio-Rad Laboratories) and the eluate was subjected to mass spectrometry using an Orbitrap Elite system at the Proteomics and Mass Spectrometry Core Facility at the University of Georgia.

### Antibodies and western blotting

Anti-SIP and anti-MST1 antibodies were generated as follows. The coding region of *SIP* was amplified by PCR from *Chlamydomonas* cDNA using primers 2 and 10 (Table S4) and cloned into the EcoRI site in the pMAL-cRI vector (New England Biolabs), downstream of the maltose-binding protein (MBP) sequence. Similarly, an ∼500-bp long stretch encoded by exon 14 of *MST1* was amplified by PCR from *Chlamydomonas* genomic DNA using primers 11 and 12 (Table S4) and inserted into the EcoR1 site of pMAL-cRI. The MBP-fusions of SIP and the MST1 fragment were expressed in *Escherichia coli* and purified using amylose resin according to the instructions of the manufacturer (New England Biolabs). Polyclonal antisera in rabbits were produced by Pocono Rabbit Farm and Laboratory and the anti-SIP antibody was affinity-purified using SIP protein immobilized on PVDF membrane.

Whole-cell samples, isolated cilia or ciliary fractions were incubated for 5 min at 95°C in Laemmli SDS sample buffer, separated by SDS-PAGE using Bio-Rad TGX precast gels, and transferred onto PVDF membrane. Membranes were blocked in TBS supplemented with 0.05% Tween 20, 3% bovine serum albumin and 3% fish gelatin followed by standard immunostaining protocols, i.e. incubation in the diluted primary antibodies for overnight at 4°C with agitation (primary antibodies used in this study are listed in Table S5) and incubation in diluted secondary antibodies (anti-mouse-IgG, 1:3000, and anti-rabbit IgG, 1:4000, conjugated to horseradish peroxidase; Invitrogen 31432/AB_228302 and 31460/AB_228341, respectively) for ∼60 min at room temperature. For visualization, membranes were incubated in chemiluminescence substrate (SuperSignal West Pico PLUS or Atto; Thermo Fisher Scientific) and the images were captured using a Bio-Rad ChemiDoc MP imaging system and the Image Lab software (Bio-Rad).

### Whole-mount negative stain EM

For whole-mount EM, a formvar- and carbon-coated 100 mesh electron microscope grid (FCF100-Cu-50, Electron Microscopy Sciences) was placed on a drop of concentrated cells (∼2×10^7^ cells/ml in water) on parafilm for 3 min. After removing excess cells using filter paper, the grid was put on a drop of 2% uranyl acetate in water for 1 to 2 min. Finally, the grid was washed with distilled water. Images were collected using a JEOL JEM1011 electron microscope. CC-620 was used as a positive control to screen for sip SIP rescue strains.

### Swimming velocity and high-speed video

To measure the swimming velocity, cells were resuspended in fresh M medium, placed in a chambered plastic slide (14-377-259; Fisherbrand), and observed using an inverted light microscope (TMS; Nikon). Images were recorded using a MU500 camera (Amscope) and the associated Topview software at a fixed exposure time of 1 s. The length of the swimming trajectories (such as those shown in Fig. 6A) were analyzed using a newly developed ‘LengthAnalysisTool’ plugin for ImageJ (the plugin is described at https://github.com/Abha99/Length-Analysis-Tool). In brief, high-contrast long-exposure images obtained using non-phototactic red light were converted into 8-bit images and analyzed using the plugin, resulting in an image in which the recognized trajectories are outlined and numbered and a pop-up table with the measurements, including the end-to-end distance representing the linear velocity of the cells and the contour length of the path representing the actual velocity (the latter was used here; Fig. S5). The annotated image and the table were examined, and false tracks were deleted. Excel was used for statistical analysis and bar graphs and violin plots were prepared using GraphPad Prism.

### *In vivo* TIRF imaging

Samples for *in vivo* imaging were prepared as follows: at room temperature, 10 μl of cells were placed inside of a ring of petroleum jelly onto a 24×60 mm no. 1.5 coverslip and allowed to settle for 1–3 min. Then, a 22×22 mm no. 1.5 coverslip with 5 μl of immobilization buffer (10 mM HEPES, 5 mM EGTA, pH 7.4) was inverted onto the larger cover glass to form a sealed observation chamber. For TIRF imaging, we used a Nikon Eclipse Ti-U inverted light microscope equipped with a 60×/1.49 NA objective lens and a 40 mW 488 nm diode laser (Spectraphysics) (Lechtreck, 2013). Images were recorded at 10 fps using the iXon X3 DU897 EMCCD camera (Andor) and the Elements software package (Nikon). ImageJ (National Institutes of Health) and the KymoResliceWide plug-in were used to analyze the recordings and generate kymograms. Kymograms, individual frames, and videos were cropped and adjusted for brightness and contrast in ImageJ and Photoshop CC 2018 (Adobe); Illustrator CC 2018 (Adobe) was used to assemble the figures. Still images mostly represent 10-frame walking averages.

For drug treatments, cells were resuspended in M medium with 20 mM mM LiCl or 20 mM NaPPi, pH 6.9; the experiments were repeated twice or more.

### High-speed video analysis

For high-speed video analysis at 1000 fps, we used an inverted Eclipse Ti2 microscope (NIKON) equipped with a long distance DIC condenser and a 40x 0.95 Planapo objective. Images were recorded using an EoSens 3CL camera (Mikrotron) and an CORE2 DVR Express rapid storage device (IO Industries). Cells were concentrated and placed in an observation chamber. Recordings were exported in AVI format and analyzed using ImageJ.

### Structure predictions

The publicly available Google ColabFold project (Mirdita et al., 2022) was used to generate structure predictions with Alphafold 2 (Jumper et al., 2021). Structure figures were generated using Pymol software.

## Supplementary Material



10.1242/joces.261497_sup1 Supplementary information
