# Supplementary material for: The Small Interactor of PKD2 protein promotes the assembly and ciliary entry of the Chlamydomonas PKD2–mastigoneme complexes
Source: J Cell Sci. 2024 Jan 12;137(1):jcs261497. doi: 10.1242/jcs.261497 (PMC10846610; doi:10.1242/jcs.261497)
Supplement: Supplementary information [file joces-137-261497-s1.pdf]

Figure S1

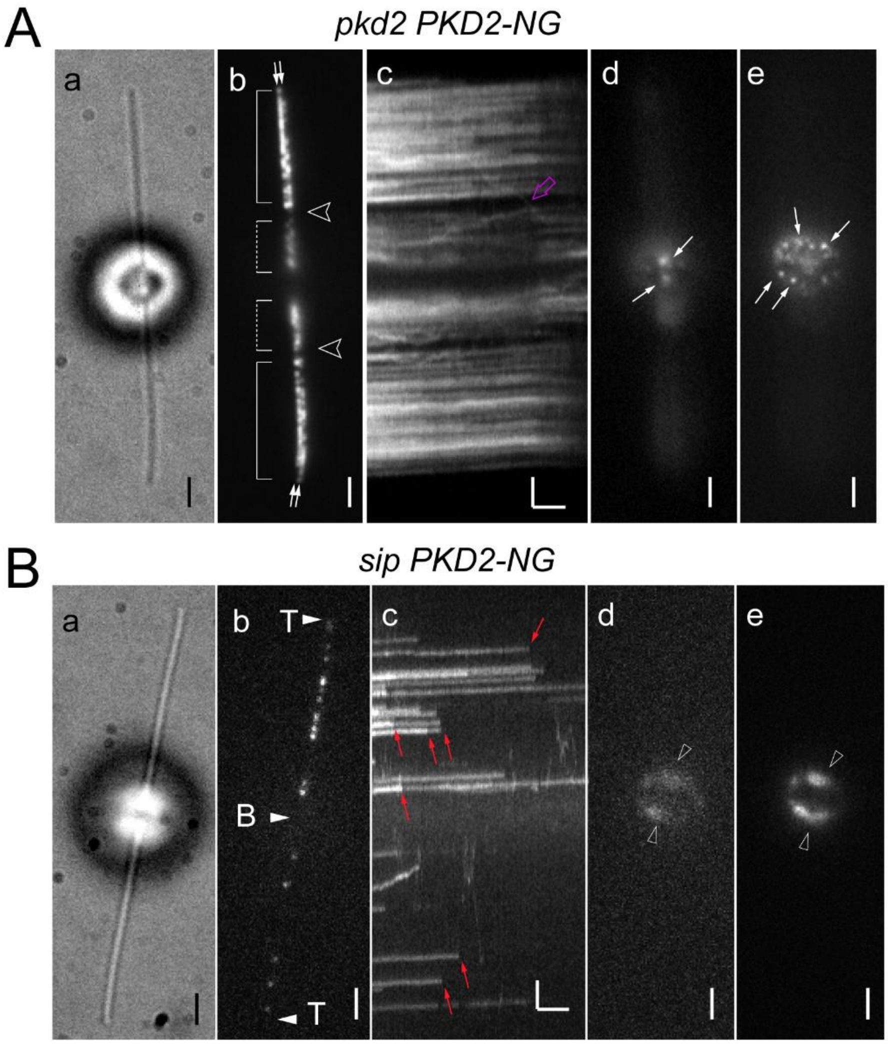

Fig. S1. The PKD2-NG cell body pool

A) Bright field (a), TIRF images (b, d, e) and corresponding kymogram (c) of a *pkd2 PKD2-NG* cell. Shown are different focal planes of the same cell, showing the adhered cilia (b), the basal body level (d) and an optical section through the cell apex (e). Arrow in c, PKD2-NG particle move through the gap. White arrows in d and e, PKD2-NG punctae in the cell body. Note dotted distribution of PKD2-NG in the latter indicative for an association with the sub-membranous microtubule cage of the cell body. Bars = 2s 2µm.

Journal of Cell Science • Supplementary information

B) Bright field (a), TIRF images (b, d, e) and corresponding kymogram (c) of a *sip PKD2-NG* cell. Shown are different focal planes of the same cell, showing the adhered cilia (b), the basal body level (d) and an optical section through the cell apex (e). Red arrows in c, bleaching events of PKD2-NG. Some particles bleached in a single step while others bleached in two steps, indicating two PKD2-NG particles. White arrowheads in d and e, autofluorescence of the plastid. Bars = 2s 2µm.

Figure S2

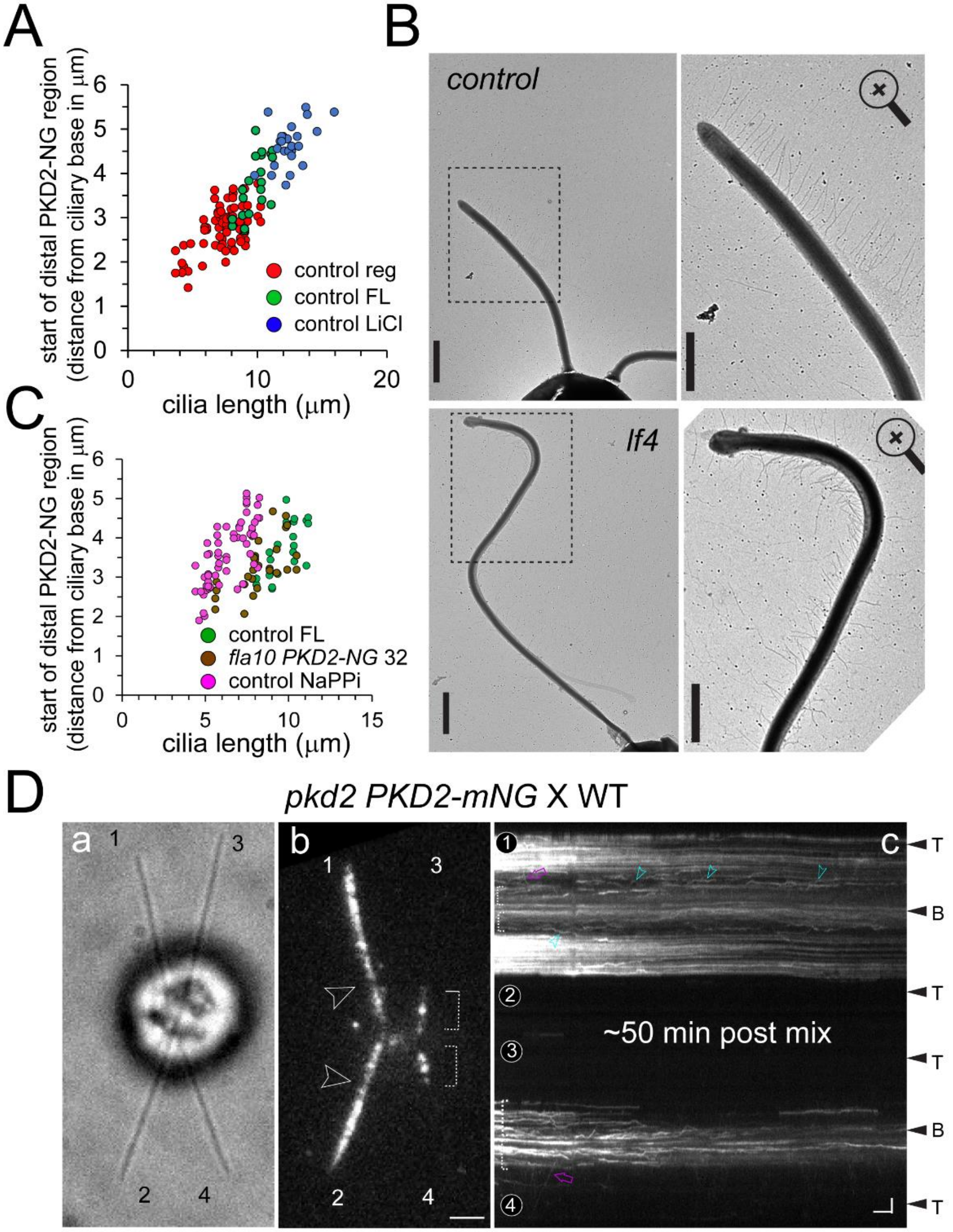

Journal of Cell Science • Supplementary information

Fig. S2. Mastigonemes are present along lf4 cilia

A) Scatter plot of the distance between the proximal border of the distal PKD2-NG region and the ciliary base versus total ciliary length. Data of control cells with regenerating (reg, red) and full-length (FL, green) cilia, and treated with LiCl (blue) are shown. Note that the distance between the proximal border of the distal region and the ciliary base increases with increasing ciliary length.

B) Whole mount EM of control (g1) and *lf4* cells. Shown are overviews (left) and details of the distal ciliary region (right). Note the presence of mastigonemes in the distal region of *lf4* cilia. C) As panel A, but showing the data for full-length and NaPPi-treated control cilia and *fla10 pkd2 PKD2-NG* cilia after incubation at 32°C.

C) Scatter plot of the distance between the proximal border of the distal PKD2-NG region and the ciliary base versus total ciliary length. Data of control cells with full-length cilia (FL, green) and treated with NaPPi (purple) and for the fla10 pkd2 PKD2-NG strain after incubation at 32°C are shown.

D) Bright field, TIRF image and corresponding kymogram of a quadriciliated *pkd2* PKD2-NG × wild-type control zygote. Cilia derived from the *pkd2 PKD2-NG* parent are labeled 1 and 2; those derived from the g1 control strain are labeled 3 and 4. The gaps, proximal regions, and ciliary tips and bases are marked. Magenta arrows, retrograde IFT; blue arrowheads, apparent diffusion of PKD2-NG. Note near absence of PKD2-NG form the distal segment of wild-type derived cilia. Bars = 2µm (in b for a and b) and 2µm 2s (c).

Journal of Cell Science • Supplementary information

Figure S3

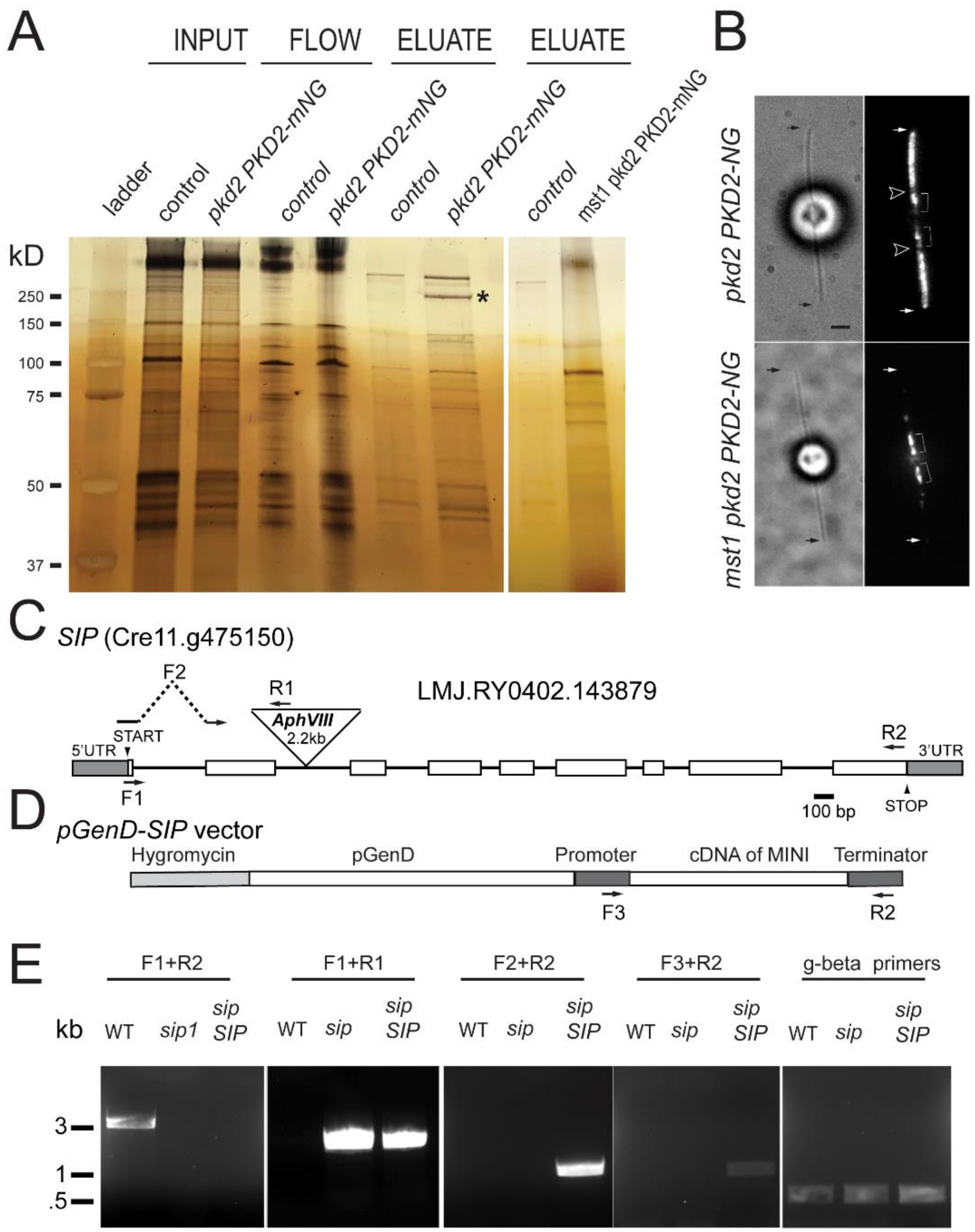

Fig. S3. Identification of SIP as a PKD2 interacting protein

A) Silver-stained gel analyzing the PKD2-NG pull-down assays from control (expressing endogenous PKD2), *pkd2 PKD2-NG* (null in endogenous PKD2 and expressing PKD2-NG) and

Journal of Cell Science • Supplementary information

mst1-1 pkd2 PKD2-NG (no mastigonemes or PKD2; expressing PKD2-NG). The lanes are showing the ciliary detergent extract (INPUT), flow through (FLOW), and eluates from the anti-NG-nanobody sepharose trap. *, putative MST1 band.

B) Bright field and TIRF images of *pkd2 PKD2-NG* and *mst1-1 pkd2 PKD2-NG* cells. The ciliary tips (small arrows) and the gaps (arrowheads) are marked. Bar = 2μm.

C) Genomic map of the *SIP*/Cre11.g475150 gene. The positions of the *AphVIII* insertion in the CLiP mutant and of the primers used to track the insertion (as shown in panel E) are indicated.

D) Schematic presentation of the cDNA-based construct used to rescue the *sip* mutant. The positions of the primers used to track the transgene are indicated.

E) Agarose gels of PCR reactions used to genotype control, *sip* mutant and *sip SIP* rescue cells.

Journal of Cell Science • Supplementary information

Figure S4

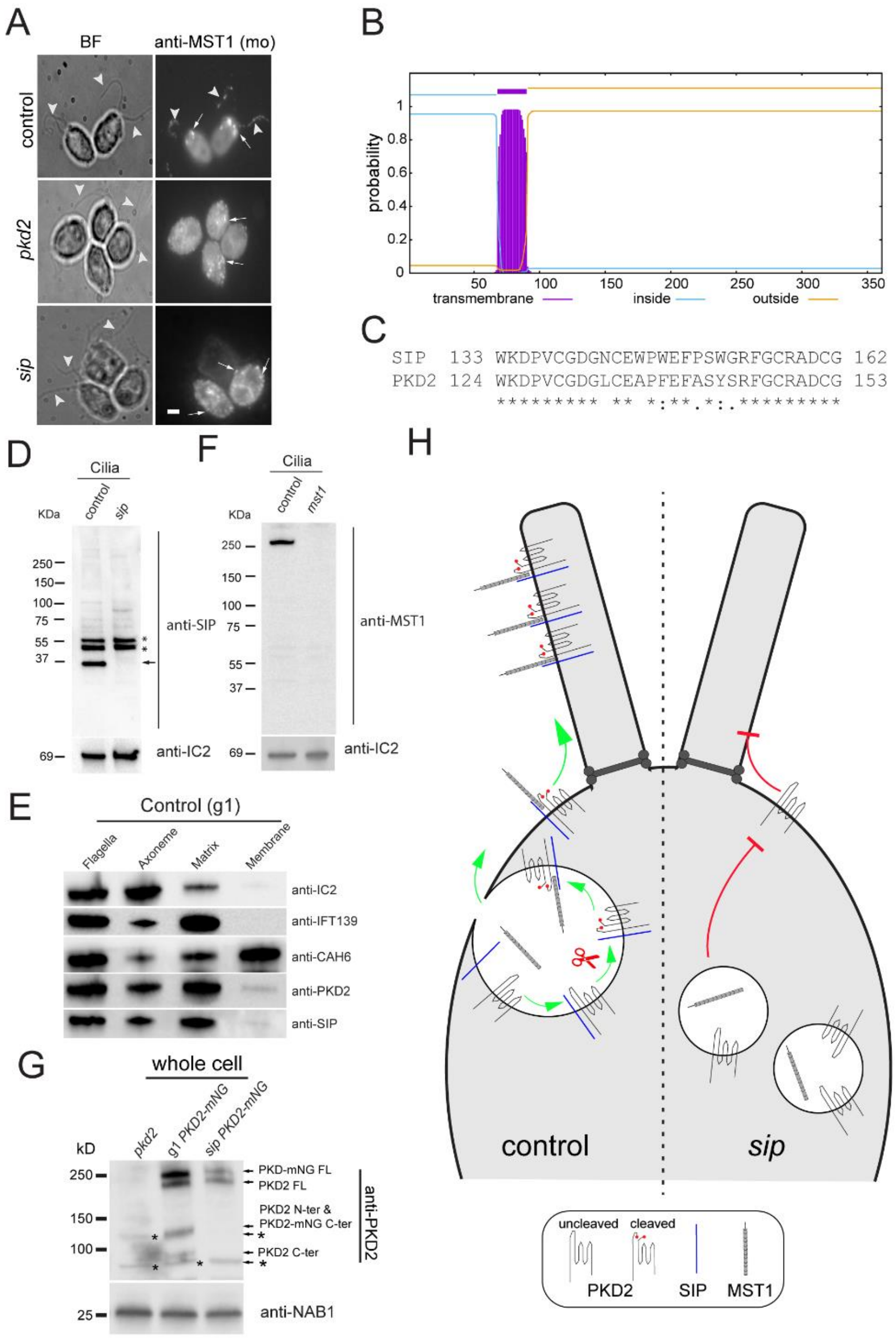

Fig. S4. Antibodies to SIP and MST1

A) Immunofluorescence staining of control, *pkd2* and *sip* mutant cells with monoclonal anti-MST1 (Nakamura et al., 1996). The pool of MST1 in control cells and dispersed MST1 spots in *pkd2* and *sip* cells are marked by arrows. Arrowheads, cilia. Bar = 2µm.

Journal of Cell Science • Supplementary information

B) Prediction of the transmembrane domain of SIP using TMHMM-2.0 at the Technical University of Denmark (https://services.healthtech.dtu.dk/services/TMHMM-2.0/). Purple, blue and orange lines indicate the sole transmembrane domain (residue 69 – 85), the intra- and the extracellular regions, respectively.

C) Conserved sequence stretches of *Chlamydomonas* PKD2 and SIP.

D) Western blot of cilia isolated from control and the *sip* mutant and probed with anti-SIP. Arrow, position of SIP; *, bands cross-reacting with anti-SIP.

E) Western blot analysis of cilia and ciliary fractions of a wild-type control strain (g1) obtained by Triton X-114 phase partitioning and stained with antibodies to SIP and antibodies to the ODA subunit IC2, the membrane-associated protein CAH6, and the IFT-B protein IFT139, respectively, as markers for the axonemal, membrane (i.e., detergent-soluble phase), and matrix (i.e., aqueous phase) fractions.

F) Western blot of cilia isolated from control and the *mst1-1* mutant probed with polyclonal anti-MST1. In D and F, anti-IC2 was used as a loading control.

G) Western blot of whole cell samples from the strains indicated probed with anti-PKD2 and anti-NAB1, as a loading control. Tagged and endogenous full-length PKD2 and the derived fragments are indicated. Note near absence of PKD2 fragments in *sip* mutant strains. *, cross-reacting unspecific bands.

H) Hypothetical model depicting assembly and targeting of the PKD2-SIP-mastigoneme complexes to cilia. In control cells, formation of the PKD2-SIP complex permits cleavage of PKD2 and sorting of vesicles to the cell apex. Then, complexes of cleaved PKD2, SIP, and mastigonemes enter the cilia. In *sip* mutants, PKD2 cleavage and interaction with the mastigonemes fails, MST1 and PKD2-containing vesicles are prevented from accumulating at the cell apex, and uncleaved PKD2 is prevented from entering cilia.

Journal of Cell Science • Supplementary information

Figure S5

A

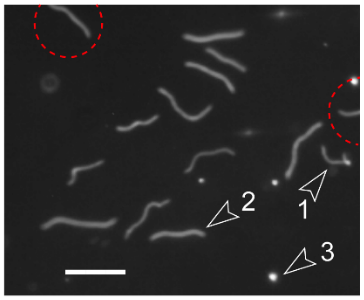

B

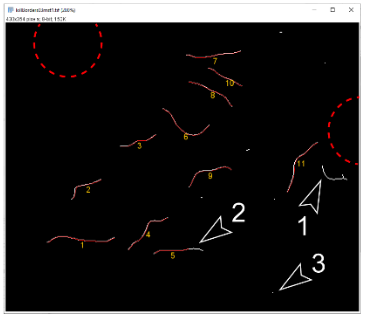

C

Results

File Edit Font Results

	Id	Length	Length in pixels	Velocity(µm/s)
1	1	81.154	87.369	119.259
2	2	41.881	47.322	64.595
3	3	44.272	48.018	65.544
4	4	61.074	66.511	90.787
5	5	41.304	42.180	57.576
6	6	59.808	77.259	105.459
7	7	65.123	69.361	94.677
8	8	59.540	64.117	87.520
9	9	54.781	63.670	86.910
10	10	45.651	48.262	65.878
11	11	69.971	76.241	104.069

Fig. S5. Analysis of swimming velocity using the Length Analysis Tool plugin in ImageJ

A) Micrograph of *mst1* cells obtained by a 1-s exposure. White lines are the trajectories of swimming cells. Bar = 100µm.

B) Result image of the Length Analysis Tool Fiji plugin. Trajectories in contact with the image border were eliminated (dashed circles). Trajectories appearing branched or crossing each other were not analyzed (arrowhead 1). Occasionally, the ends of trajectories were not correctly

Journal of Cell Science • Supplementary information

recognized by the plugin (arrowhead 2). Non-moving cells (arrowhead 3) were not considered by the plugin as the trajectory length is below the set threshold.

C) Corresponding result table. Length, end-to-end length; Length in pixels, actual length of the path. The velocity data are based on the length-in-pixels measurement.

Journal of Cell Science • Supplementary information

Table S1. Mass spectrometry of PKD2-NG immune-isolates

List of proteins identified by mass spectrometry of PKD2-NG complexes pulled-down from the *pkd2 PKD2-NG* and *mst1-1 pkd2 PKD2-NG* strains. The protein names, Phytozome accession numbers, hits (peptides identified in x of n experimental samples), coverage, total and unique peptides, and the ratio between the coverage in two experimental strains are indicated. The following proteins were found in all preparations, including those from the untagged control strain: FMG-1, FMG-1b, FAP33, FAP12, beta-tubulin, cobalamin-independent methionine synthase, eukaryotic translation elongation factor 1 alpha 1, putative blue light receptor, alpha-tubulin, IFT88, S-Adenosyl homocysteine hydrolase, IFT72/74, IFT81, IFT80, IFT57. The * indicates proteins, for which putative or confirmed mutants were obtained from the Chlamydomonas Resource Center; of these strains, only the *sip* mutant lacked mastigonemes.

protein	link	MW	***pkd2 PKD2-NG*** (4 repeats)				***mst1 pkd2 PKD2-NG*** (3 repeats)				Ratio of coverage
			hits	Coverage (average %)	Total peptides (average #)	Unique peptides (average #)	hits	Coverage (%)	Total peptides (average #)	Unique peptides (average #)	(*pkd2 PKD2-NG/mst1 pkd2 PKD2-NG*)
PKD2	Cre17.g715300	1626	4/4	11.8	40.2	19.5	3/3	8.9	45	15	1.33
MST1	Cre16.g650600	1987	4/4	6.2	23.7	9.7	0/3	0	0	0	-
FAP48	Cre16.g665450	3140	2/4	1.4	7.5	4	3/3	5	52.7	15.3	0.28
FAP24*	Cre02.g081050	537	3/4	5	5.5	2.7	2/3	6.2	8.3	2.7	0.8
enolase	Cre12.g513200	477	4/4	8.7	7.2	3.7	3/3	13.6	18	3.3	0.64
IFT172	Cre17.g703900	1755	2/4	2.5	9.7	4.2	3/3	7.6	46.3	13.7	0.33
FAP154	Cre08.g362100	4441	2/4	0.7	7.2	2.7	3/3	3.4	42	12	0.2
ODA5-associated AK	Cre01.g029750	560	2/4	3.7	4.7	1.2	3/3	14.9	22	4.3	0.25
IFT144	Cre02.g095072	1321	2/4	1.4	4.5	2	3/3	6.3	28.3	8.3	0.22
FAP39	Cre02.g145100	930	2/4	2.7	3.5	2	2/3	3.2	12	2.7	0.83
FAP10*	Cre12.g505350	1009	2/4	3.1	5.5	3	3/3	7.2	21.3	6.7	0.43
SIP*	Cre11.g475150	361	4/4	9.4	6.7	3.5	1/3	4.7	5	1.7	2
EF-2	Cre17.g737	845	1/	1.2	2.5	1	3/	5	13.7	4	0.23

Journal of Cell Science • Supplementary information

	250		4				3				
FAP5	Cre12.g518550	1347	1/4	1	2.2	1.5	3/3	5.8	28	8.3	0.18
FAP208*	Cre11.g482001	1373	1/4	0.7	2	1	3/3	2.4	10.7	3	0.31
IFT38	Cre17.g721250	325	1/4	2.2	1.5	0.7	3/3	10.4	10.6	4	0.37
IFT139	Cre06.g268800	1311	2/4	1.4	3	2	3/3	3.7	16.3	5.7	0.37
FAP295/PRKG2*	Cre16.g663200	718	1/4	1.3	1	0.7	3/3	13.8	53.3	11.3	0.09
Chlorophyll a/b BP	Cre01.g066917	253	1/4	1.6	1	0.5	1/3	2.5	5.3	0.7	0.63
IFT122	Cre01.g065822	1239	1/4	1.4	2.5	1.5	3/3	2.8	14.3	4	0.48
phosphoglyceromutase	Cre06.g272050	557	1/4	1.4	2	1	2/3	5	11.7	3.3	0.29
IFT70	Cre07.g342200	647	1/4	0.66	1	0.5	3/3	5.1	12.3	4.3	0.13
actin (IDA5)*	Cre13.g603700	377	3/4	5.44	4	2	3/3	14.2	22.7	5.6	0.38
FAP148	Cre10.g434600	1732	1/4	0.55	1.5	1	1/3	1.8	9.3	3.3	0.29
FAP333 (ARF)	Cre12.g486250	181	1/4	3.6	1.5	0.5	3/3	17.3	12.3	2.7	0.21
EF-3	Cre04.g222700	1053	2/4	2	3.5	2.25	3/3	3.9	11.7	4.7	0.51
FAP252/BUG1	Cre01.g065822	352	1/4	1.6	1	0.5	1/3	3	3	1	0.51
psbC	Cre10.g466650	461	2/4	3.1	2.7	1.2	2/3	5.1	10	2	0.6
FAP19/CGK2	Cre02.g076900	1027	1/4	0.5	0.7	0.5	2/3	5.6	22.7	5.7	0.09

Journal of Cell Science • Supplementary information

Table S2. Distribution and Conservation of SIP in green algae

The *C. reinhardtii* sequences for SIP, PKD2, and MST1 and Uniprot Blast were used search for related sequences of each protein in green algae. In contrast to mammalian PKD2 homologues, green algal PKD2 possess a large insertion in the extracellular loop 1. The UniProt accession numbers, or, if those were not available, the NCBI accession numbers, are shown.

	SIP (361)		MST1 (1987)		PKD2 (1644)	
Species	Length/Accession #	identity	Length/Accession #	identity	Length/Accession #	identity
Chlamydomonas incerta	368/A0A835SNE0	95.1	1955/A0A835TMQ	55.8	1643/A0A835TGR6	94.5
Gonium pectorale	359/A0A150GFF9	82.7	868/KXZ54501	52.8	1629/A0A150GMD 5	76.6
Volvox carteri f. nagariensis	405/D8U8N9	74	835/XP_002946994	51.5	1606/D8U257	67.1
Trebouxia sp. A1-2	478/KAA6421199	58.7	n.d.	-	1618/ A0A5J4YB07	48.9
Chlorella sorokiniana	476/A0A2P6TP89	47.7	n.d.	-	1689/A0A2P6TS23	39.4
*Scenedesmus sp. NREL 46B-D3 **	300/A0A7J7Q6W8	47	n.d.	-	1582/ A0A7J7Q397	43
Dunaliella salina	360/KAF5841058	37.5	n.d.	-	1784/ KAF5834587	47.4

Journal of Cell Science • Supplementary information

Table S3. Strains used in this study.

**Name**	**Genoytpe**	**Reference**
CC-620 (wild-type)	*Nit, nit2,mt*$^{+}$	*Chlamydomonas* Genetics Centre
g1 (wild-type)	*nit1, agg1, mt*$^{+}$	(Pazour et al., 1995)
CC-5325 (wild-type)	*cw15, mt-*	(Li et al., 2016)
pkd2 (progeny selected from *pkd2*^{CLiP} backcrossed twice with *g1*)	*cw15, pkd2, mt*$^{-}$	(Liu et al., 2020)
lf4 (CC-4768)	*lf4, mt*$^{+}$	(Berman et al., 2003)
fla10 (CC-1919)	*fla10-1, mt-*	*(Huang et al., 1977)*
pkd2 PKD2-NG (CC-5899)	*pkd2, PKD2-NG, mt*$^{-}$	This study
pkd2 PKD2-GFP	*pkd2, PKD2-GFP, mt*$^{+}$	(Liu et al., 2020)
sip (LMJ.RY0402.143879)	*sip, cw15, mt*$^{-}$	(Li et al., 2019)
sip SIP	*sip, SIP, cw15,mt*$^{-}$	This study
sip PKD2-NG	*sip PKD2-NG, mt-*	This study
mst1-1 (LMJ.RY0402.052413)	*mst1, cw15,mt*$^{-}$	(Li et al., 2019; Liu et al., 2020)
mst1-2 (LMJ.RY0402.136134)	*mst1, cw15, mt*$^{-}$	(Amador et al., 2020; Li et al., 2019)

Journal of Cell Science • Supplementary information

Table S4. Primers used in this study

Primer number	Primer name	Sequence (5' to 3')
1	SIP cloning F	5'CGCCATATGGCAGAACAACACCCGAG3'
2	SIP cloning R	5'CGCGAATTCTTACACCGCCGCGGTTCCC3
3	Hyg Hind	5'CGCAAGCTTGTTTCTTGCGCTATGACACTTG3'
4	Hyg	5'CGCAAGCTTCGCTTCAAATA3'
5	GENO SIP	5'CGCGGATCCATGGCAGAACGTGAGTCGCCT3'
6	JONI	5'TGTCGCTGAAAGTGGAGGTC3'
7	pGenDf	5'TGCTCGGGGGGAGGTTTCCT3'
8	g-beta f1	5'CAAGCTGAAGAACAACCTGGTG3'
9	g-beta r1	5'CTTGCTGGTGATGTTGAACTCG3'
10	SIP anti F	5'CGCGAATTCATGGCAGAACAACACCCGAG3'
11	MSTf ANTI	5'CGCGAATTCGTGTCAACTTCTGGCGCTAC3'
12	MSTr ANTI	5'CGCGAATTCTCACTGGCACGCAGTGGCACC3'

Table S5. Antibodies used in this study.

Name	Host	Dilution WB	Dilution IF	Reference
anti-PKD2	rb	1:2000		(Huang et al.,2007)
anti-SIP	rb	1:2000		This study
anti-MST1	rb	1:2000		This study
anti-MST1	mo		1:200	(Nakamura et al., 1996)
anti-IC2	mo	1:1000		(King and Witman, 1990)
anti-CAH6	rb	1:500		(Yu et al.,2020)
anti-IFT139	mo	1:100		(Cole et al., 1998)

Journal of Cell Science • Supplementary information

References

Cole, D. G., Diener, D. R., Himelblau, A. L., Beech, P. L., Fuster, J. C. and Rosenbaum, J. L. (1998). Chlamydomonas kinesin-II-dependent intraflagellar transport (IFT): IFT particles contain proteins required for ciliary assembly in Caenorhabditis elegans sensory neurons. *J Cell Biol* **141**, 993-1008.

Huang, B., Rifkin, M. R. and Luck, D. J. (1977). Temperature-sensitive mutations affecting flagellar assembly and function in Chlamydomonas reinhardtii. *J Cell Biol* **72**, 67-85.

King, S. M. and Witman, G. B. (1990). Localization of an intermediate chain of outer arm dynein by immunoelectron microscopy. *J Biol Chem* **265**, 19807-19811.

Li, X., Zhang, R., Patena, W., Gang, S. S., Blum, S. R., Ivanova, N., Yue, R., Robertson, J. M., Lefebvre, P. A., Fitz-Gibbon, S. T. et al. (2016). An Indexed, Mapped Mutant Library Enables Reverse Genetics Studies of Biological Processes in Chlamydomonas reinhardtii. *Plant Cell* **28**, 367-387.

Yu, K., Liu, P., Venkatachalam, D., Hopkinson, B. M. and Lechtreck, K. F. (2020). The BBSome restricts entry of tagged carbonic anhydrase 6 into the cis-flagellum of Chlamydomonas reinhardtii. *PLoS One* **15**, e0240887.

Journal of Cell Science • Supplementary information
