# Supplementary material for: The Small Interactor of PKD2 protein promotes the assembly and ciliary entry of the Chlamydomonas PKD2–mastigoneme complexes
Source: J Cell Sci. 2024 Jan 12;137(1):jcs261497. doi: 10.1242/jcs.261497 (PMC10846610; doi:10.1242/jcs.261497)
Supplement: Supplementary file 1 [file joces-137-261497_review_history.pdf]

Journal of Cell Science

The Small Interactor of PKD2 protein promotes the assembly and ciliary entry of the *Chlamydomonas* PKD2-mastigoneme complexes

Poulomi Das, Betlehem Mekonnen, Rama Alkhofash, Abha V. Ingle, E. Blair Workman, Alec Feather, Gui Zhang, Nathan Chasen, Peiwei Liu and Karl F. Lechtreck
DOI: 10.1242/jcs.261497

Editor: David Stephens

Review timeline
Original submission: 20 July 2023
Editorial decision: 4 September 2023
First revision received: 23 November 2023
Accepted: 4 December 2023

Original submission

<u>First decision letter</u>

MS ID#: JOCES/2023/261497

MS TITLE: Small Interactor of PKD2 (SIP), a novel single-pass transmembrane protein, is required for proteolytic processing and ciliary import of Chlamydomonas PKD2

AUTHORS: Poulomi Das, Betlehem Mekonnen, Rama Alkhofash, Abha Vivek Ingle, Elizabeth Blair Workman, Alec Feather, Gui Zhang, Peiwei Liu, and Karl Lechtreck
ARTICLE TYPE: Research Article

We have now reached a decision on the above manuscript.

To see the reviewers' reports and a copy of this decision letter, please go to: https://submit-jcs.biologists.org and click on the 'Manuscripts with Decisions' queue in the Author Area. (Corresponding author only has access to reviews.)

As you will see, the reviewers raise a number of substantial criticisms that prevent me from accepting the paper at this stage. They suggest, however, that a revised version might prove acceptable, if you can address their concerns. If you think that you can deal satisfactorily with the criticisms on revision, I would be pleased to see a revised manuscript. We would then return it to the reviewers.

I agree with their conclusions that some further data analysis would greatly strengthen the manuscript, notably around the focus on the absolute distance between the base of the cilia and the proximal portion. I consider that their comments are very clear and hope that you will be able to incorporate them into a revised version. I can also see the added value of some AlphaFold modelling and hope that you will be able to consider including this.

Please ensure that you clearly highlight all changes made in the revised manuscript. Please avoid using 'Tracked changes' in Word files as these are lost in PDF conversion.

I should be grateful if you would also provide a point-by-point response detailing how you have dealt with the points raised by the reviewers in the 'Response to Reviewers' box. Please attend to all of the reviewers' comments. If you do not agree with any of their criticisms or suggestions please explain clearly why this is so.

© 2023. Published by The Company of Biologists under the terms of the Creative Commons Attribution License (https://creativecommons.org/licenses/by/4.0/).

Reviewer 1

Advance summary and potential significance to field

1. Strong use of proteomics and Chlamydomonas genetics to define a new protein SIP and characterisation of its mutant strain.

2. Proposes a novel non-signalling role for PKD2 channel in Chlamydomonas i.e., attachment of mastigonemes to distal ciliary compartment to enhance swimming velocity.

3. Interesting findings on ciliary patterning, organisation, and scaling of sub-ciliary membrane domains. This could be of general interest to ciliary cell biologists.

Comments for the author

Major comments:

1. Inclusion of some findings (PKD2 ectosome shedding) is not clearly linked or relevant to the main points of the study which are the PKD2 proximo-distal patterning and discovery plus speculated roles of SIP in PKD2 processing and formation of PKD2-mastigoneme complexes. This section could be better integrated and explained.

2. Please explicitly state in the text the exact size/length of the proximal PKD2 domain in microns (ex. x +/- x um) instead of ratios in relation to the distal zone and/or flagellar length. Such proximal domains are common to other (membrane/soluble?) ciliary proteins (ex. ODA10 3-4 um), and it would be helpful to have an exact value for the length of this domain for easy comparison.

3. Please clarify if mastigoneme density/number scales with flagellar length when the distal domain expands in the lf4 mutants? This would be another strong evidence to link PKD2 functions in mastigoneme attachment.

4. As this study describes a novel protein SIP, it would be helpful to include an Alphafold predicted structure (this is readily available via EBI) and SIPs domain organisation highlighting its TMD. This would support the discussion point about single-pass membrane proteins.

5. Unless already mentioned, UniProt IDs for the other proteins described (ex. PKD2, MST1) would also be useful in Table S2.

Minor comments:

1. Although discussed briefly, this reviewer would encourage additional discussion on the nature of the gap/boundary between proximal and distal ciliary PKD2 domains. There are a number of open questions such as below:

o Does this proximo-distal partitioning extend to other membrane or soluble proteins in Chlamydomonas and other systems?

o Is the lipid composition and therefore mobility of membrane proteins in the exclusion zone distinct? Are there studies on this? This relates to a broader point which needs to be discussed about the equally intriguing dorso-ventral patterning of PKD2 in nodal cilia.

o What is the role of IFT versus diffusion (or both) to cross this gap, which appears to be a Chlamydomonas specific second diffusion barrier in an otherwise contiguous organelle.

2. A more explicit general commentary/sub-section on the reliance of various ciliary proteins on IFT or free diffusion (or the need for both) to cross the various diffusion zones to find their

© 2023. Published by The Company of Biologists under the terms of the Creative Commons Attribution License (https://creativecommons.org/licenses/by/4.0/).

ciliary sites of action inside/on the Chlamydomonas flagella (and cilia of other systems) is highly encouraged.

Reviewer 2

Advance summary and potential significance to field

The polycystic disease protein, PKD2, functions in the cilia of unicellular and multicellular organisms. In male nematodes, its disruption leads sensory behavioral defects. In humans, its mutation leads to polycystic kidney disease, one of the most common inherited diseases in humans, as well as to left-right laterality defects. PKD2 has been proposed to have receptor-like properties as well as channel properties, and is implicated in ciliary signaling and calcium homeostasis, but many details of PKD2 molecular function remain unknown. This manuscript addresses the properties of PKD2 in the cilia of the bi-ciliated, green alga Chlamydomonas.

The authors build on their important 2020 findings on Chlamydomonas ciliary PKD2. They showed that PKD2 interacts with a subset of outer doublet microtubules on the distal 2/3 of the ciliary axoneme. They also reported that PKD2 is essential to anchor a motility-related protein (MST1) polymer on the extracellular side of the membrane to carry out PKD2's function in ciliary motility, as the pkd2 mutant also exhibited reduced swimming velocity.

Here, in studies on the ciliary localization of PKD2, the authors show that the PKD2-MST1 complex is dynamically associated with the axoneme during ciliogenesis, and that as cilia grow, previously anchored PKD2-MST1 complexes that were most proximal to the ciliary base are selectively released from the axoneme, as new complexes are added at the tip of the growing microtubule doublets. Once ciliogenesis is complete, the PKD2-MST1 complexes remain relatively stable. The second major and more mechanistic finding of the manuscript is that appearance of the mature, cleaved form of PKD2 in cilia requires a newly identified protein, SIP. In the absence of SIP, PKD2 fails to undergo proteolytic cleavage, ciliary amounts of PKD2 are substantially reduced, and MST1 fails to be anchored. Given that PKD2 in other organisms also undergoes proteolytic cleavage and is also localized at discrete sites on cilia, these finding are of potentially broad relevance.

Comments for the author

As written, the manuscript, including the additional finding that PKD2 can be shed from the tips and bases of cilia and the confirmation of previous results that MST1 polymers are essential for wild-type motility, lacks a mechanistic focus and is presented as almost a natural history of PKD2 in Chlamydomonas. More detailed comments are below.

Introduction lacks key information and coherence: For the reader to appreciate the importance of these investigations, the Introduction should provide more context and a fuller description of functional relevance. It was challenging to determine what was known previously and what is being investigated in this new manuscript. It was also difficult to understand the major gaps in knowledge of Chlamydomonas PKD2 that were being investigated here. The current evidence indicates that the sole function of PKD2 in Chlamydomonas is to anchor MST1 polymers (mastigonemes) and thereby regulate cell motility, yet in the Introduction, mastigonemes are added as an afterthought at the end of a sentence, with no mention of their function in motility. In addition, the focus of the Introduction is on ciliary sub-compartments, yet the title indicates that the work focuses on proteolytic processing of PKD2, even though no mention is made in the Introduction that PKD2 is known to be proteolytically processed in Chlamydomonas and other organisms.The authors should also consider adding here information that is now only in the Discussion, that in 2020 another group reported that cell motility was unaffected by the loss of mastigonemes.

It could be argued that one of the most important as yet unanswered questions about PKD2 in Chlamydomonas is whether the motility phenotype of the pkd2 mutants is a consequence simply of the loss of MST1, or whether the absence of PKD2 channel function also contributes to the motility phenotype.

© 2023. Published by The Company of Biologists under the terms of the Creative Commons Attribution License (https://creativecommons.org/licenses/by/4.0/).

That is, does a mutant form of PKD2 that lacks channel function still anchor mastigonemes and still regulate motility. The Introduction (or the Discussion?) could indicate that the studies reported here provide a foundation for addressing this other important question.

Consistency between Fig. 1, Fig. 2, and text: Measurement of the brackets in Fig. 1A shows that the distal and proximal regions of the full length cilia have a distal/proximal (d/p) ratio of 3, yet the text at the beginning of Results states the ratio for wild-type full length cilia is 2 (2/3 : 1/3). Also, Fig. 2A graph shows the average ratio for full length cilia is 2, and none of the data points are at 3? If the Fig. 1A image is non-representative, the authors should so indicate.

The grey boxes in Fig. 2B are labeled “control (reg and FL).” “Control” seems to be a misnomer, since “reg” presumably stands for “regenerating,” thus, an experimental set of data. Also, do the FL (presumably full length?) points represent values from cilia that had reached full length at the end of regeneration, or were they from a different sample of cells with steady state cilia, or were they from cilia of the experimental cells before their cilia were detached?

Need to present original length data used to calculate the distal/proximal ratios: Text on p. 6 describes distal/proximal (d/p) ratios for cilia below 5 μm, for cilia greater than 5 μm, and for full length cilia, but the Fig. 2A X-axis does not depict length. Rather it depicts ciliary regeneration time. Text and figures need to be consistent. Examination of Fig. 2 B, which does show d/p ratio vs length, suggests that arbitrarily dividing the samples into lengths less than or equal to 5 μm and lengths greater than 5 μm might not be useful. Rather, examination gives the impression that the d/p ratios fell into 3 categories during regeneration. For cilia lengths of 1-4 μm, the d/p ratio appeared to be around 2, not 2.5 as suggested in the text. For the larger group of samples clustered around 5 μm, the d/p ratio appeared to be closer to 2.8 or 3. And at lengths greater than ~7 μm, the d/p ration settled back to ~2.

One of the most exciting findings in the manuscript! If a more thorough examination of the regeneration data in Fig. 2 B along with statistical analysis support the impressions described above that the d/p ratio for cilia less than ~4 um is around 2, I think that this would be strong evidence of a proportionate increase in lengths of the two compartments during ciliary regeneration. As indicated in the Discussion, the only way this could happen is if PKD2-MST1 complexes that initially were anchored close to the ciliary base early during regeneration are subsequently removed as the cilia grow in length. I am not aware of other work that presents evidence on changes in localization of microtubule-anchored and membrane-anchored ciliary proteins during ciliary growth. Thus, I feel that this evidence for dynamic regulation of axonemal composition is one of the most exciting findings in the manuscript. Currently, this concept of dynamic regulation is completely buried in the Results and mentioned briefly in the Discussion. The authors should consider revising the way they framed the results to emphasize that the PKD2-mastigoneme complex must be highly dynamic.

Additionally, I think a case could be made that the manuscript would have more impact if all the findings that now focus on the d/p ratio instead focused on the absolute distance between the base of the cilia and the proximal portion of the distal region and its changes during ciliogenesis. I do not think that the authors sufficiently and convincingly justified the importance of determining the pattern of development of the two regions? What functional or structural insights will come from studying the pattern as opposed to studying the absolute locations? If the authors think that cells can sense pattern in the context of ciliary length, they need to present a stronger case.

More information needed for the ciliary shortening experiments: Perusal of the 1978 Lefebvre paper showed that cells in NaPPI resorbed their cilia to ~50% of their original length in 60 minutes, and to ~10% of their original length in 120 minutes. If the kinetics were similar here, it is difficult to be convinced that grouping the lengths into a single average length (~6.3) for the 60 minute time and the 120 minute time has biological meaning. Indeed, examination of the Fig. 2B NaPPi results gives the impression that the d/p ratio for cilia that had shorted to lengths ~4 - 6 μm was below 2, whereas that of longer cilia was ~2. If so, then in the cilia that had shortened the most, only the distal PKD2 region might have shortened, which is different from the conclusion in the text. I should note, though, that it was difficult to visualize all of the data points for the shortening expreiments, and my impression might well be incorrect. As indicated above for the growth

© 2023. Published by The Company of Biologists under the terms of the Creative Commons Attribution License (https://creativecommons.org/licenses/by/4.0/).

experiments, for the reader to be able to evaluate these shortening results, the lengths and distances from the cilia base of the borders of both PKD2 regions need to be presented, not just the ratios. More data points might be needed to fully assess changes in PKD2 regions during shortening.

p. 8; Need to provide the d/p ratio for fla10 cells at the permissive temperature before being placed at the restrictive temperature. Fig. 2 legend needs to indicate that the fla10 cells were at the restrictive temperature (if correct?).

Justification of PKD2 release results: The experiments on release of PKD2 are intriguing but they don't seem to fit in with the previous or following experiments? Need to provide a rationale. What question was being asked?

Discussion: p. 19; Using the word "size" here implies that absolute lengths of the two regions are the focus of this manuscript, but absolute lengths were rarely presented. Rather the data are presented as ratios, without, as indicated above, providing the reader with the rationale for focusing on ratios rather than absolute lengths.

Need a transition to the reader understand the connection between PKD2 localization and the new PKD2-related protein, SIP.

SIP results: This part of the manuscript is mechanistic and compelling. It could be further strengthened by use of AI-based structural analysis methods to provide a more thorough assessment of SIP. Within 15-30 minutes of using AlphaFold and related analysis methods, I found the SIP structure predicted by AlphaFold (AF-A8JFQ9-F1-model_v4.pdb) and used FoldSeek to search for related proteins. In addition to several other hits, the Candida, Dictyostelium, and human hits in the CATH50 FoldSeek search results contained a fold that is also found in several proteases, including in the human meprin metalloprotease sheddase:
“Structural basis for the sheddase function of human meprin β metalloproteinase at the plasma membrane (https://doi.org/10.1073/pnas.1211076109). Of course, this initial, potentially intriguing result might not stand up to more a more robust analysis. Given the ease of obtaining potentially useful structural information, however, about this newly identified protein, and if the cursory look described above is confirmed, such an analysis would add depth to the current manscript.

Minor points:

p. 6; Need to tell reader what PKD2-NG is. The abbreviation is in the Abbreviations section, but it should also be presented on first use. Reader will not understand the meaning of "distinct motility." The motility needs to be described in the text.

p. 7: Wondering is not a compelling rationale for doing experiments.

Typos and other editing mistakes: The text need to be carefully checked for typos, mistaken use of tenses, and lack of subject-verb agreement.

First revision

Author response to reviewers' comments

Response to the referees’ comments

We appreciate the referees’ thorough work and constructive critiques. We agree with the vast majority of the comments and have revised the manuscript accordingly.

Reviewer 1 Advance summary and potential significance to field
1.Strong use of proteomics and Chlamydomonas genetics to define a new protein SIP and characterisation of its mutant strain.

© 2023. Published by The Company of Biologists under the terms of the Creative Commons Attribution License (https://creativecommons.org/licenses/by/4.0/).

2.Proposes a novel non-signalling role for PKD2 channel in Chlamydomonas i.e., attachment of mastigonemes to distal ciliary compartment to enhance swimming velocity.

3.Interesting findings on ciliary patterning, organisation, and scaling of sub-ciliary membrane domains. This could be of general interest to ciliary cell biologists.

Reviewer 1 Comments for the author
Major comments:

1.Inclusion of some findings (PKD2 ectosome shedding) is not clearly linked or relevant to the main points of the study which are the PKD2 proximo-distal patterning and discovery plus speculated roles of SIP in PKD2 processing and formation of PKD2-mastigoneme complexes. This section could be better integrated and explained.

This concern was also raised by referee 2. We agree that this section stands alone and has little relevance to the remainder of the manuscript. We therefore removed this section from the revised manuscript and focused on the two main points, i.e., development of PKD2-containing ciliary regions and characterization of the SIP protein.

2.Please explicitly state in the text the exact size/length of the proximal PKD2 domain in microns (ex. x +/- x um) instead of ratios in relation to the distal zone and/or flagellar length. Such proximal domains are common to other (membrane/soluble?) ciliary proteins (ex. ODA10 3-4 um), and it would be helpful to have an exact value for the length of this domain for easy comparison.

This was also requested by referee 2. We agree that providing the original length data is required and revised the manuscript and Figures 2 and S2 accordingly. We also added a supplementary table allowing for a side-by-side comparison of the measurements.

3.Please clarify if mastigoneme density/number scales with flagellar length when the distal domain expands in the lf4 mutants? This would be another strong evidence to link PKD2 functions in mastigoneme attachment.

We performed whole mount negative staining EM of the lf4 mutant. Indeed, mastigonemes cover the distal region of lf4 cilia. The data were added to Supplementary Figure S2 and mentioned in the text. While not analyzed in detail, the density of mastigonemes on lf4 cilia was comparable to that observed in control cilia.

4.As this study describes a novel protein SIP, it would be helpful to include an Alphafold predicted structure (this is readily available via EBI) and SIPs domain organisation highlighting its TMD. This would support the discussion point about single-pass membrane proteins.

Alphafold documentation of SIP was also requested by referee 2. In the revised manuscript, Fig. 4B shows the Alphafold2 predictions for SIP and PKD2. The figure highlights the structural similarity between the two proteins.

5.Unless already mentioned, UniProt IDs for the other proteins described (ex. PKD2, MST1) would also be useful in Table S2.

When available, Uniprot accession numbers were added to Table S2.

Minor comments:

1.Although discussed briefly, this reviewer would encourage additional discussion on the nature of the gap/boundary between proximal and distal ciliary PKD2 domains. There are a number of open questions such as below:

Does this proximo-distal partitioning extend to other membrane or soluble proteins in Chlamydomonas and other systems?

© 2023. Published by The Company of Biologists under the terms of the Creative Commons Attribution License (https://creativecommons.org/licenses/by/4.0/).

To our best knowledge this is the first description of a partitioning of a channel protein into regions with distinct properties. In the discussion and introduction, we describe relevant other examples of ciliary subdomains. .

Is the lipid composition and therefore mobility of membrane proteins in the exclusion zone distinct? Are there studies on this? This relates to a broader point which needs to be discussed about the equally intriguing dorso-ventral patterning of PKD2 in nodal cilia.

While it is well established that the lipid composition of cilia is distinct from that of the plasma membrane, specific lipid domains within the cilium of Chlamydomonas have, to my knowledge, not described in a definitive manner. An exception is the detergent-insoluble membrane which covers the transition zone of Chlamydomonas. In the introduction, it is now mentioned that PKD2 has a preferred localization on one side of the nodal cilia in mice, namely the side facing the nodal flow.

What is the role of IFT versus diffusion (or both) to cross this gap, which appears to be a Chlamydomonas specific second diffusion barrier in an otherwise contiguous organelle.

PKD2-NG crosses the gap between the proximal and distal region by both IFT and diffusion. Such events are now marked in the kymograms shown in Figs. 1, 5, and S1. In the revised text, we state: "The proximal and distal PKD2-NG regions are typically (>90% of cilia) separated by a more or less conspicuous gap of ~1 µm length lacking PKD2 but for the occasional particle passing through by IFT or diffusion (Figs. 1C, S1A)"
In the discussion, we offer two possible explanations for the presence of a gap and discuss intraciliary barriers in other systems. Without additional knowledge of the molecular nature of the gap, I feel that not much more can be added.

2.A more explicit general commentary/sub-section on the reliance of various ciliary proteins on IFT or free diffusion (or the need for both) to cross the various diffusion zones to find their ciliary sites of action inside/on the Chlamydomonas flagella (and cilia of other systems) is highly encouraged.

IFT of tagged PKD2 was not observed in C. elegans and our data suggest that IFT has a limited role in transporting PKD2-NG into Chlamydomonas cilia. We mention the role of IFT in maintaining the subciliary distribution of GCY-22, a ciliary membrane protein in the introduction. With respect to PKD2-NG, we show that the role of IFT in establishing its ciliary distribution is unclear. Because the discussion is already quite long and the manuscript is at the length limit of the journal, a detailed review of the topic is not possible.

Reviewer 2 Advance summary and potential significance to field
The polycystic disease protein, PKD2, functions in the cilia of unicellular and multicellular organisms. In male nematodes, its disruption leads sensory behavioral defects. In humans, its mutation leads to polycystic kidney disease, one of the most common inherited diseases in humans, as well as to left-right laterality defects. PKD2 has been proposed to have receptor-like properties as well as channel properties, and is implicated in ciliary signaling and calcium homeostasis, but many details of PKD2 molecular function remain unknown. This manuscript addresses the properties of PKD2 in the cilia of the bi-ciliated, green alga Chlamydomonas.

The authors build on their important 2020 findings on Chlamydomonas ciliary PKD2. They showed that PKD2 interacts with a subset of outer doublet microtubules on the distal 2/3 of the ciliary axoneme. They also reported that PKD2 is essential to anchor a motility-related protein (MST1) polymer on the extracellular side of the membrane to carry out PKD2's function in ciliary motility, as the pkd2 mutant also exhibited reduced swimming velocity.

Here, in studies on the ciliary localization of PKD2, the authors show that the PKD2-MST1 complex is dynamically associated with the axoneme during ciliogenesis, and that as cilia grow, previously anchored PKD2-MST1 complexes that were most proximal to the ciliary base are selectively released from the axoneme, as new complexes are added at the tip of the growing microtubule doublets. Once ciliogenesis is complete, the PKD2-MST1 complexes remain relatively stable. The second major and more mechanistic finding of the manuscript is that appearance of the mature,

© 2023. Published by The Company of Biologists under the terms of the Creative Commons Attribution License (https://creativecommons.org/licenses/by/4.0/).

cleaved form of PKD2 in cilia requires a newly identified protein, SIP. In the absence of SIP, PKD2 fails to undergo proteolytic cleavage, ciliary amounts of PKD2 are substantially reduced, and MST1 fails to be anchored. Given that PKD2 in other organisms also undergoes proteolytic cleavage and is also localized at discrete sites on cilia, these finding are of potentially broad relevance.

Reviewer 2 Comments for the author
As written, the manuscript, including the additional finding that PKD2 can be shed from the tips and bases of cilia and the confirmation of previous results that MST1 polymers are essential for wild-type motility, lacks a mechanistic focus and is presented as almost a natural history of PKD2 in Chlamydomonas. More detailed comments are below.

Introduction lacks key information and coherence: For the reader to appreciate the importance of these investigations, the Introduction should provide more context and a fuller description of functional relevance. It was challenging to determine what was known previously and what is being investigated in this new manuscript. It was also difficult to understand the major gaps in knowledge of Chlamydomonas PKD2 that were being investigated here. The current evidence indicates that the sole function of PKD2 in Chlamydomonas is to anchor MST1 polymers (mastigonemes) and thereby regulate cell motility, yet in the Introduction, mastigonemes are added as an afterthought at the end of a sentence, with no mention of their function in motility.

We agree and extended the introduction section to provide information on PKD2 in various systems. We also include more background on the Chlamydomonas PKD2-MST1 complex.

In addition, the focus of the Introduction is on ciliary sub-compartments, yet the title indicates that the work focuses on proteolytic processing of PKD2, even though no mention is made in the Introduction that PKD2 is known to be proteolytically processed in Chlamydomonas and other organisms.The

We agree and now mention that Chlamydomonas PKD2 is cleaved in the revised introduction. In contrast to PKD1/polycystin-1, proteolytic cleavage of PKD2 has to our knowledge, not been reported in other systems.

The authors should also consider adding here information that is now only in the Discussion, that in 2020 another group reported that cell motility was unaffected by the loss of mastigonemes.

In the meantime, a novel study focusing on mastigonemes ultrastructure, also reported near normal motility for the mst1 mutant. Both studies are not cited in the introduction as well as in the discussion section.

Both studies are now discussed and cited in the introduction and the discussion.

It could be argued that one of the most important as yet unanswered questions about PKD2 in Chlamydomonas is whether the motility phenotype of the pkd2 mutants is a consequence simply of the loss of MST1, or whether the absence of PKD2 channel function also contributes to the motility phenotype. That is, does a mutant form of PKD2 that lacks channel function still anchor mastigonemes and still regulate motility. The Introduction (or the Discussion?) could indicate that the studies reported here provide a foundation for addressing this other important question.

The need to find means to study PKD2 channel function independently of mastigonemes, e.g., via PKD2 gain-of-function and loss-of-function constructs, is now discussed (p.25): "As PKD2 is reduced in mst1 cilia and pkd2 cilia lack mastigonemes, assigning individual role to each protein is difficult. Loss-of-function and gain-of-function PKD2 mutants, which still bind mastigonemes and the axoneme, could allow for a better assessment of the putative channel function of Chlamydomonas PKD2."

Consistency between Fig. 1, Fig. 2, and text: Measurement of the brackets in Fig. 1A shows that the distal and proximal regions of the full length cilia have a distal/proximal (d/p) ratio of 3, yet the text at the beginning of Results states the ratio for wild-type full length cilia is 2 (2/3 : 1/3). Also, Fig. 2A graph shows the average ratio for full length cilia is 2, and none of the data points are at 3? If the Fig. 1A image is non-representative, the authors should so indicate.

© 2023. Published by The Company of Biologists under the terms of the Creative Commons Attribution License (https://creativecommons.org/licenses/by/4.0/).

We agree that the original manuscript failed to properly provide and discuss data on the length of the length of the PKD2 region in cilia. This part of the manuscript was substantially revised. We removed the 1/3 to 2/3 description and provide the actual lengths measurements of the PKD2-NG regions. We also added a table providing an overview of the various strains and conditions (Table 1).

For control cells, the average ratio is 2.3 (distal:proximal). The cell shown in Fig. 1 is close to that range; the image was chosen as it allows one to recognize the two rows of PKD2-NG.

The grey boxes in Fig. 2B are labeled “control (reg and FL).” “Control” seems to be a misnomer, since “reg” presumably stands for “regenerating,” thus, an experimental set of data. Also, do the FL (presumably full length?

We apologize for not providing a better description of these data. The revised figure legend clarifies that “control” refers to the pkd2 PKD2-NG rescue strain (to set it apart from strains carrying additional mutations); “FL” and “reg” are now also explained in the figure legend.

points represent values from cilia that had reached full length at the end of regeneration, or were they from a different sample of cells with steady state cilia, or were they from cilia of the experimental cells before their cilia were detached?

Full-length refers to cilia of untreated cells without prior deciliation step. This is now mentioned in the text. We avoid using the term “steady state” as, in our hands, full-length cilia of vegetatively grown Chlamydomonas are quite static with little tubulin turnover at the tip (Harris et al. MBoC 2016). Once regenerating cilia reach full length, we refer to them as “fully regenerated”. Such cilia were not analyzed in this study.

Need to present original length data used to calculate the distal/proximal ratios: Text on p. 6 describes distal/proximal (d/p) ratios for cilia below 5 μm, for cilia greater than 5 μm, and for full length cilia, but the Fig. 2A X-axis does not depict length. Rather it depicts ciliary regeneration time. Text and figures need to be consistent. Examination of Fig. 2 B, which does show d/p ratio vs length, suggests that arbitrarily dividing the samples into lengths less than or equal to 5 μm and lengths greater than 5 μm might not be useful. Rather, examination gives the impression that the d/p ratios fell into 3 categories during regeneration. For cilia lengths of 1-4 μm, the d/p ratio appeared to be around 2, not 2.5 as suggested in the text. For the larger group of samples clustered around 5 μm, the d/p ratio appeared to be closer to 2.8 or 3. And at lengths greater than ~7 μm, the d/p ration settled back to ~2.

This point was also raised by referee 1 and we agree that a revision is required. We now show the actual length of the PKD2 region. We eliminated the subdivision of regrowing cilia (i.e., into below/above 5 um or by time) as this doesn’t adds to the conclusions. The manuscript was rewritten to focus on the main point that the proximal and distal PKD2 regions develop proportionally until cilia reach full-length and that it is predominately the distal PKD2 region which increases in abnormally long cilia.

One of the most exciting findings in the manuscript! If a more thorough examination of the regeneration data in Fig. 2 B along with statistical analysis support the impressions described above that the d/p ratio for cilia less than ~4 um is around 2, I think that this would be strong evidence of a proportionate increase in lengths of the two compartments during ciliary regeneration. As indicated in the Discussion, the only way this could happen is if PKD2-MST1 complexes that initially were anchored close to the ciliary base early during regeneration are subsequently removed as the cilia grow in length. I am not aware of other work that presents evidence on changes in localization of microtubule-anchored and membrane-anchored ciliary proteins during ciliary growth. Thus, I feel that this evidence for dynamic regulation of axonemal composition is one of the most exciting findings in the manuscript. Currently, this concept of dynamic regulation is completely buried in the Results and mentioned briefly in the Discussion. The authors should consider revising the way they framed the results to emphasize that the PKD2-mastigoneme complex must be highly dynamic.

© 2023. Published by The Company of Biologists under the terms of the Creative Commons Attribution License (https://creativecommons.org/licenses/by/4.0/).

We appreciate the referee's comments on the value of these data. Point taken that we failed to properly describe the underlying data and highlight the conclusions. We think that the revised manuscript, particularly Fig. 2E and Fig. S2, displays the data in a better way to support our conclusion that the proximal and distal PKD2 regions develop proportionally with ciliary length. In this figure, we show that

Additionally, I think a case could be made that the manuscript would have more impact if all the findings that now focus on the d/p ratio instead focused on the absolute distance between the base of the cilia and the proximal portion of the distal region and its changes during ciliogenesis. I do not think that the authors sufficiently and convincingly justified the importance of determining the pattern of development of the two regions? What functional or structural insights will come from studying the pattern as opposed to studying the absolute locations? If the authors think that cells can sense pattern in the context of ciliary length, they need to present a stronger case.

I like this idea. In the new Figs. S2 A and C, we plot the distance of the proximal border of the distal PKD2-NG region from the ciliary base vs. total length of cilia. The plots illustrate nicely that the proximal border of the distal region shifts toward the ciliary tip with increasing ciliary length and shifts toward the ciliary base when cilia shorten. The figures are introduced in the text.

In the new Table 1, we now also list the length of the gap between the proximal and distal PKD2 regions.

As ciliary PKD2-NG is distributed into stripes and distinct regions along the proximo-distal axis and these two zones apparently scale with respect to ciliary length, the term patter seems appropriate.

More information needed for the ciliary shortening experiments: Perusal of the 1978 Lefebvre paper showed that cells in NaPPI resorbed their cilia to ~50% of their original length in 60 minutes, and to ~10% of their original length in 120 minutes. If the kinetics were similar here, it is difficult to be convinced that grouping the lengths into a single average length (~6.3) for the 60 minute time and the 120 minute time has biological meaning.

For this experiment, cells were observed during a 60-minute time window starting 1 hour after the addition of NaPPi. In the results section, we state: "After a 60 to 120-minute incubation in 20 mM NaPPi, most cells (~90%) had partially resorbed their cilia to an average length of ~6.4μm (Fig. 2A-C, D d, e, Table S1)."

Indeed, examination of the Fig. 2B NaPPi results gives the impression that the d/p ratio for cilia that had shorted to lengths ~4 - 6 μm was below 2, whereas that of longer cilia was ~2. If so, then in the cilia that had shortened the most, only the distal PKD2 region might have shortened, which is different from the conclusion in the text. I should note, though, that it was difficult to visualize all of the data points for the shortening expreiments, and my impression might well be incorrect. As indicated above for the growth experiments, for the reader to be able to evaluate these shortening results, the lengths and distances from the cilia base of the borders of both PKD2 regions need to be presented, not just the ratios. More data points might be needed to fully assess changes in PKD2 regions during shortening.

We did this analysis (see panel on the left. Open squares, length of distal region; filled squares, length of proximal region). While the data are noisy, the overall tendency suggests that both regions decrease in length as cilia get shorter (see also Fig. 2).

p. 8; Need to provide the d/p ratio for fla10 cells at the permissive temperature before being placed at the restrictive temperature. Fig. 2 legend needs to indicate that the fla10 cells were at the restrictive temperature (if correct?).

This is indeed an important control. We now include measurements on fla10 PKD2-NG cells obtained at the permissive temperature. (see Fig. 2).

Justification of PKD2 release results: The experiments on release of PKD2 are intriguing but they don't seem to fit in with the previous or following experiments? Need to provide a rationale. What question was being asked?

© 2023. Published by The Company of Biologists under the terms of the Creative Commons Attribution License (https://creativecommons.org/licenses/by/4.0/).

This point was also raised by referee 1. We agree that the data on the gliding cells are insufficiently related to the rest of the manuscript and decided to omit this part in the revised manuscript.

Discussion: p. 19; Using the word "size" here implies that absolute lengths of the two regions are the focus of this manuscript, but absolute lengths were rarely presented. Rather the data are presented as ratios, without, as indicated above, providing the reader with the rationale for focusing on ratios rather than absolute lengths.

In the revised manuscript, the absolute lengths of the PKD2 regions are provided (Fig. 2A, B, Table 1). The term “size” was replaced with length throughout the manuscript and usage of the terms “ratio” and “pattern” was reduced.

The ratios are now shown in Fig. 2 panel C. This figure also shows why ratios can be useful in interpreting the data: While ratios of ~2.3 between the distal and proximal PKD2 regions were observed for all strains and conditions regardless of ciliary length as long as it is below that of standard full-length cilia (~10 um), distinct ratios are observed when cilia exceed the standard length as observed for lf4 and LiCl treated cells.

Need a transition to the reader understand the connection between PKD2 localization and the new PKD2-related protein, SIP.

We added an introductory sentence to the section on discovery of SIP. The flow of the text is also improved by the removal of the section on gliding cells.

SIP results: This part of the manuscript is mechanistic and compelling. It could be further strengthened by use of AI-based structural analysis methods to provide a more thorough assessment of SIP. Within 15-30 minutes of using AlphaFold and related analysis methods, I found the SIP structure predicted by AlphaFold (AF-A8JFQ9-F1-model_v4.pdb) and used FoldSeek to search for related proteins. In addition to several other hits, the Candida, Dictyostelium, and human hits in the CATH50 FoldSeek search results contained a fold that is also found in several proteases, including in the human meprin metalloprotease sheddase: “Structural basis for the sheddase function of human meprin β metalloproteinase at the plasma membrane (https://doi.org/10.1073/pnas.1211076109). Of course, this initial, potentially intriguing result might not stand up to more a more robust analysis. Given the ease of obtaining potentially useful structural information, however, about this newly identified protein, and if the cursory look described above is confirmed, such an analysis would add depth to the current manscript.

The Alphafold2 structures of SIP and PKD2 have been added to Fig 4 of the revised manuscript. The referee is correct that SIP shares some folds with certain proteases. However, on further analysis, it appears that the similarity to proteases does not involve the protease domains themselves. For example, the similarity with Meprin is in Meprin's MAM domain (aa335-aa400) and the overlap with the calpain protease is in an unannotated region (aa504-aa574) outside of the peptidase domain. Taken together, we think that it is highly unlikely that SIP is a protease for PKD2. This point of view has been added to the revised discussion.

Minor points:
p. 6; Need to tell reader what PKD2-NG is. The abbreviation is in the Abbreviations section, but it should also be presented on first use.

Yes. Done.

Reader will not understand the meaning of "distinct motility." The motility needs to be described in the text.

During the revision, the phrase was rewritten.

p. 7: Wondering is not a compelling rationale for doing experiments.

© 2023. Published by The Company of Biologists under the terms of the Creative Commons Attribution License (https://creativecommons.org/licenses/by/4.0/).

The phrase was rewritten and details on the motility were provided.

Typos and other editing mistakes: The text need to be carefully checked for typos, mistaken use of tenses, and lack of subject-verb agreement.

The text was edited by a native English speaker.

Second decision letter

MS ID#: JOCES/2023/261497

MS TITLE: Small Interactor of PKD2 (SIP) promotes the assembly and ciliary entry of the Chlamydomonas PKD2-mastigoneme complexes

AUTHORS: Poulomi Das, Betlehem Mekonnen, Rama Alkhofash, Abha Vivek Ingle, Elizabeth Blair Workman, Alec Feather, Gui Zhang, Nathan Chasen, Peiwei Liu, and Karl Lechtreck
ARTICLE TYPE: Research Article

I am happy to tell you that your manuscript has been accepted for publication in Journal of Cell Science, pending standard ethics checks. (I am confident that the minor typos will be corrected in proofing).

Reviewer 1

Advance summary and potential significance to field

1. Strong use of proteomics and Chlamydomonas genetics to define a new protein SIP and characterisation of its mutant strain.

2. Proposes a novel non-signalling role for PKD2 channel in Chlamydomonas i.e., attachment of mastigonemes to distal ciliary compartment to enhance swimming velocity.

3. Interesting study on ciliary patterning, organisation, and scaling of sub-ciliary membrane domains. This could be of general interest to ciliary cell biologists

Comments for the author

The authors have addressed the major comments to a high level of detail.
- They have now provided a detailed quantification of the original length data which was requested.
- They have also shown by wholemount negative stain EM that mastigonemes are present along the longer lf4 mutant flagella.
- They have removed the section on PKD2 ectosome shedding. It now makes the manuscript more cohesive and may help readers. This reviewer hopes the authors are able to incorporate the ectosome shedding data into another separate manuscript.
- They have included AlphaFold2 predictions for SIP and PKD2 in Figure 4 and highlighted some minor structural similarities.

As for minor points, the additional inclusion of the requested discussion points and clarification of the roles of IFT versus diffusion is appreciated.

Overall, the authors have addressed all the points raised by this reviewer. No further revisions are suggested. Subject to reviewer 2’s points being addressed to their satisfaction, this reviewer would recommend that this very nice manuscript be accepted for publication.

© 2023. Published by The Company of Biologists under the terms of the Creative Commons Attribution License (https://creativecommons.org/licenses/by/4.0/).

Reviewer 2

Advance summary and potential significance to field

Please see my review of the first manuscript version.

Comments for the author

The authors have addressed my concerns, and the manuscript is appropriate for publication. This revised version is much more well integrated and unified, and provides new ideas and insights about regulation of PKD2 and ciliary patterning and about mastigoneme function.

I really like Fig. 2E. Even without any text, it tells a strong story about the dynamic features of the two ciliary domains.

I noticed several minor typos in the discussion.

© 2023. Published by The Company of Biologists under the terms of the Creative Commons Attribution License (https://creativecommons.org/licenses/by/4.0/).
